# Rational engineering of *Kluyveromyces marxianus* to create a chassis for the production of aromatic products

**DOI:** 10.1186/s12934-020-01461-7

**Published:** 2020-11-11

**Authors:** Arun S. Rajkumar, John P. Morrissey

**Affiliations:** School of Microbiology, Centre for Synthetic Biology and Biotechnology, Environmental Research Institute, APC Microbiome Institute, University College Cork, Cork, T12 K8AF Ireland

**Keywords:** 2-phenylethanol, Aromatic amino acid, Shikimate pathway, Pentose phosphate pathway, Phosphoketolase, PEP synthase

## Abstract

**Background:**

The yeast *Kluyveromyces marxianus* offers unique potential for industrial biotechnology because of useful features like rapid growth, thermotolerance and a wide substrate range. As an emerging alternative platform, *K. marxianus* requires the development and validation of metabolic engineering strategies to best utilise its metabolism as a basis for bio-based production.

**Results:**

To illustrate the synthetic biology strategies to be followed and showcase its potential, we describe a comprehensive approach to rationally engineer a metabolic pathway in *K. marxianus*. We use the phenylalanine biosynthetic pathway both as a prototype and because phenylalanine is a precursor for commercially valuable secondary metabolites. First, we modify and overexpress the pathway to be resistant to feedback inhibition so as to overproduce phenylalanine de novo from synthetic minimal medium. Second, we assess native and heterologous means to increase precursor supply to the biosynthetic pathway. Finally, we eliminate branch points and competing reactions in the pathway and rebalance precursors to redirect metabolic flux to a specific product, 2-phenylethanol (2-PE). As a result, we are able to construct robust strains capable of producing over 800 mg L^−1^ 2-PE from minimal medium.

**Conclusions:**

The strains we constructed are a promising platform for the production of aromatic amino acid-based biochemicals, and our results illustrate challenges with attempting to combine individually beneficial modifications in an integrated platform.

## Introduction

Microbial cell factories are an important part of an emerging sustainable bioeconomy. By metabolically engineering microbial hosts, it is possible to synthesise chemical compounds that are currently sourced from non-renewable resources or from renewable resources that may not meet the growing demands of the chemical, food and pharmaceutical industries. The process typically involves cloning heterologous pathways for such compounds into host microbes and altering native pathways to ensure that metabolism is optimised for the products of interest. The budding yeast *Saccharomyces cerevisiae* has proved to be an excellent host for de novo synthesis of valuable secondary metabolites in the flavonoid, stilbenoid and alkaloid families [1]. Several of these are directly derived from aromatic amino acids: flavonoids, stilbenoids and aroma compounds from phenylalanine [2]; benzylisquinoline alkaloids from tyrosine [3]; and monoterpene alkaloids from tryptophan [4]. Others are derived from intermediates of the shikimate pathway, such as the flavour compound vanillin [5] and the fine chemical muconic acid [6]. Optimising precursor supply is of special interest if these compounds are to be produced at titres for commercial production competitive with existing commercial sources. As a result, the metabolic engineering of *S. cerevisiae* to overproduce aromatic amino acids (AAAs) has been the subject of considerable research and development to produce diverse secondary metabolites of commercial value [1, 7]. Furthermore, phenylalanine and tyrosine are commercial products in themselves; at present they are among the few amino acids that are not predominantly industrially produced by bacterial fermentation on an industrial scale [8].

**Fig. 1:**
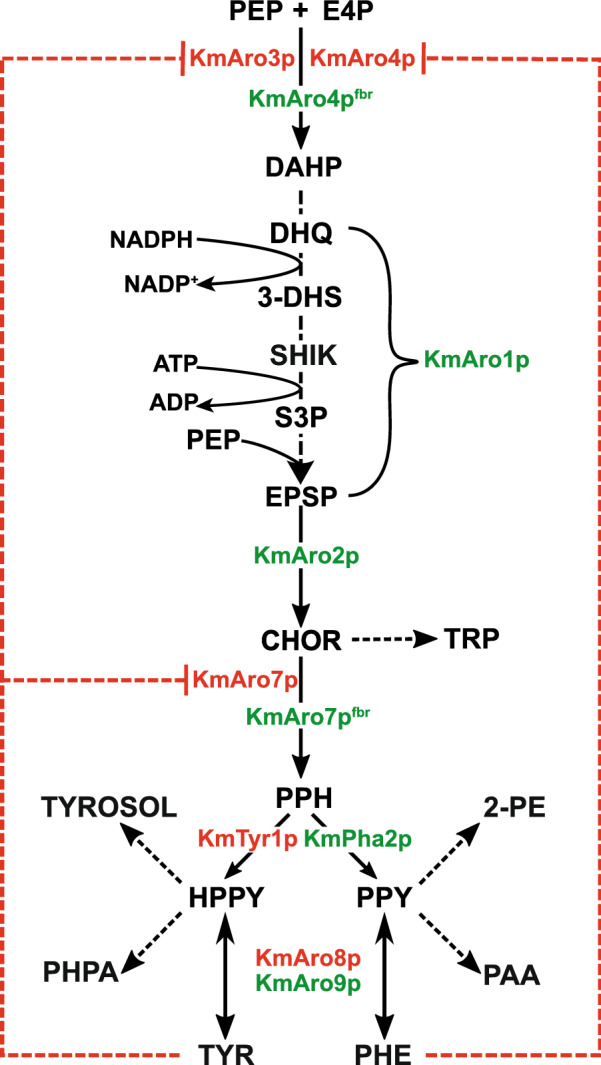
Shikimate and phenylalanine/tyrosine biosynthesis in *Kluyveromyces marxianus*. The enzymes are named according to their orthologues in *S. cerevisiae*. In our study, genes for enzymes coloured red have been deleted or otherwise inactivated and those coloured green are overexpressed, with feedback-resistant alleles represented by the superscript fbr. Red dashed lines indicate product inhibition on enzymes, while dashed arrows indicated multiple reactions between the metabolites indicated. Abbreviations: PEP: phosphoenolpyruvate, E4P: erythrose 4-phosphate, DAHP: 3-deoxy-D-arabinoheptulosonate-7-phosphate, DHQ: 3-dehydroquinate, 3-DHS: 3-dehydroshikimate, SHIK: shikimic acid, S3P: shikimate-3-phosphate EPSP: 5-enoylpyruvateshikimate-3-phosphate, CHOR: chorismate, PPH; prephentate, (H)PPY: (hydroxyl)phenylpyruvate, 2-PE: 2-phenylethanol; PAA: phenylacetic acid; TYROSOL: tyrosol/para-hydroxyphenylethanol; PHPA: para-hydroxphenylacetate; TRP: tryptophan; TYR: tyrosine, PHE: phenylalanine

In yeast, AAA synthesis begins with the shikimate pathway (Fig. 1), wherein the DAHP synthases Aro3p and Aro4p convert erythrose-4-phosphate (E4P) and phosphoenolpyruvate (PEP) to 3-deoxy-D-arabinoheptulosonate-7-phosphate (DAHP). This is in turn converted to chorismate via Aro1p and Aro2p. Chorismate is then either committed to phenylalanine/tyrosine biosynthesis via conversion to prephenate by Aro7p, or to tryptophan biosynthesis via conversion to anthranilate by Trp2p/Trp3p*.* In the former case, the enzymes Pha2p or Tyr1p convert prephenate to phenyl- or hydroxyphenyl-pyruvate, which are then transaminated to phenylalanine or tyrosine respectively by Aro8p and Aro9p. In native biosynthetic pathways, Aro3p, Aro4p and Aro7p are feedback inhibited by tyrosine and/or phenylalanine as committing to this pathway is metabolically expensive. To overcome this limitation, feedback-resistant alleles of all three genes have been identified and exploited to overproduce AAAs. Typically, overexpressing feedback-resistant (fbr) *ARO4* (*ARO4*^*fbr*^) and *ARO7* (*ARO7*^*fbr*^) is sufficient to overproduce phenylalanine and tyrosine for the production of secondary metabolites [2, 9]. Beyond this, various studies have partially or completely overexpressed the shikimate or phenylalanine/tyrosine pathways to further direct flux down this pathway. For example, E4P supply to the pathway can be increased in *S. cerevisiae* by partially overexpressing the non-oxidative branch of its pentose phosphate pathway (PPP) [6, 10] and PEP supply to the shikimate pathway has been increased by enhancing its natural production or slowing its conversion to pyruvate [11, 12].

While *S. cerevisiae* remains a favoured host, for several reasons, there is considerable value in exploring alternative yeast platforms for the synthesis of aromatic compounds. First, the native metabolism of *S. cerevisiae* has evolved for ethanol production, often at the expense of other pathways. Thus, the metabolism of a Crabtree-negative yeast can offer a better starting point since carbon flux is not diverted to ethanol and the carbon distribution between the various pathways that give rise to anabolic precursors is better balanced. Second, some non-*Saccharomyces* yeasts possess advantageous traits such as rapid growth, thermotolerance, salt tolerance, or the capacity to use a wider array of substrates [13]. The development of alternative hosts has previously been hampered by insufficient knowledge of cellular physiology, limited genetic tools, and a lack of experience of developing scaled bioprocesses. The rapid growth in the implementation of omics technologies, coupled with the development of synthetic biology [14], is helping address the first two of these problems, and several non-*Saccharomyces* yeasts are close to crossing the threshold to becoming credible first-choice hosts for defined applications.

One such yeast is the Crabtree-negative *Kluyveromyces marxianus*. It can tolerate temperatures over 50 °C and has the fastest growth rate of any eukaryote [15, 16]. It can natively consume pentose sugars and disaccharides found in agricultural and dairy wastes, making these economic feedstocks for potential cell factories. The past decade has seen development of tools for its metabolic engineering such as CRISPR/Cas9 systems, libraries of regulatory elements, secretory peptides, and in vivo assembly protocols [17]*.* In parallel, *K. marxianus* has been successfully used as a platform to produce heterologous compounds such as short-chain alcohols, carotenoids and polyketides [18–20]. However, all the latter studies focus on the overexpression of heterologous enzymes or pathways and only a few studies attempt to engineer metabolism to improve production [21, 22]. With regards to aromatic compounds, *K. marxianus* has been evolved to overproduce phenylalanine and by extension the aromatic alcohol 2-phenylethanol (2-PE) via an enhanced Ehrlich pathway [23], but this has shed little light on appropriate metabolic engineering strategies. As its physiology relies more on respiration than that of *S. cerevisiae*, approaches to metabolically engineer *K. marxianus* to maximise precursor supply and product titres and yields can vary significantly from known ones in baker’s yeast, especially since the production of aromatics is not a fermentative process.

In this work, we adopt a structured approach to metabolically engineer *Kluyveromyces marxianus’* AAA biosynthetic pathway to overproduce phenylalanine. Our strategy follows three stages. First, we alleviate feedback inhibition of the native shikimate pathway to establish AAA overproduction, followed by overexpression of the phenylalanine biosynthetic enzymes to enhance the production of phenylalanine. Second, we attempt to increase the supply of E4P and PEP to the shikimate pathway, first independently and then simultaneously, using native and heterologous enzymes. Finally, we redirect flux to phenylalanine synthesis alone by knocking down competing synthetic and degradative pathways. These strategies were assessed individually and in combination, and multiple different strategies aimed at similar goals were evaluated. Following this strategy allowed us to increase and redirect flux to the phenylalanine pathway so that it comprises up to 90% of the aromatic products synthesised. In the course of our work, we track the production of 2-phenylethanol (2-PE), the Ehrlich degradation alcohol of phenylalanine as a proxy for the production of this amino acid. Our systematic investigation of engineering strategies for phenylalanine overproduction provide a roadmap for future metabolic engineers and synthetic biologists wishing to work with *K. marxianus*.

## Results

### Establishing feedback-resistant phenylalanine biosynthesis in *Kluyveromyces marxianus*

The aromatic amino acid biosynthetic and degradative pathways in *K. marxianus* share all steps in common with *S. cerevisiae* (Fig. 1; Additional file 1: Table S1), though there are some differences in numbers of paralogous genes. While *S. cerevisiae* has three phenylpyruvate decarboxylases Pdc5p, Pdc6p and Aro10p, *K. marxianus* only has a homologue to Aro10p and to Pdc5p*,* whose enzymatic activity is uncharacterised [24]. In contrast, besides homologues of the two aminotransferases found in *S. cerevisiae* (*ARO8* and *ARO9*), sequenced *K. marxianus* genomes contain a third gene annotated as another putative aminotransferase [25]. The rational synthetic biology approach that we adopted involved the construction and analysis of over 60 strains in a complex matrix as we tested ways to enhance and optimise flux through the AAA biosynthetic pathway. A chart that shows the most important strains in this matrix is provided to aid understanding of the metabolic engineering approach and the strains generated in this study (Additional file 1: Fig. S1).

**Fig. 2:**
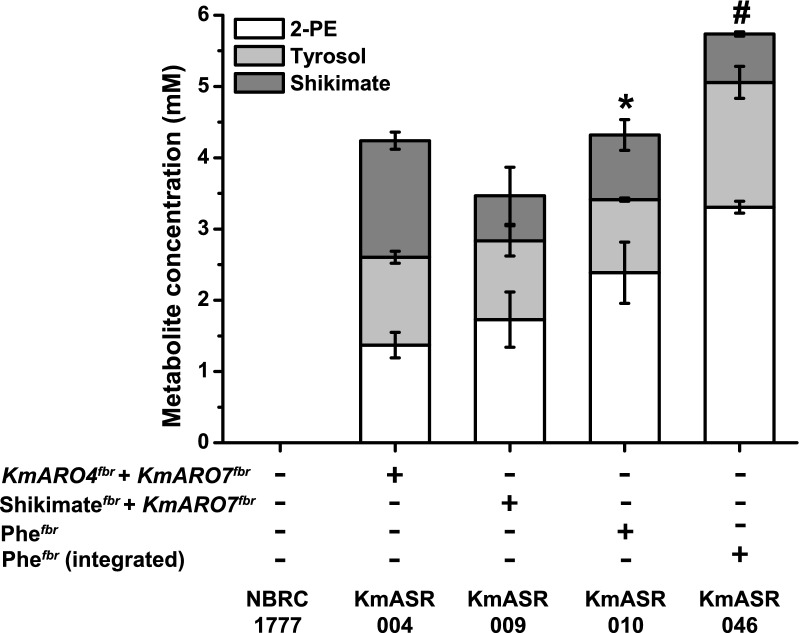
Overexpression of a feedback-resistant phenylalanine biosynthetic pathway. The two feedback-resistant alleles of *KmARO4* and *KmARO7* constructed based on homology to *S. cerevisiae* are functional, with their episomal overexpression leading to the production of Ehrlich alcohols of phenylalanine (white) and tyrosine (grey) from the overflow of their increased synthesis, as well as excess shikimate (dark grey). All compounds were measured by HPLC in culture medium after 48 h growth at 30 °C as described in the Methods. Subsequent co-expression of the shikimate (*KmARO4*^*fbr*^*, KmARO1, KmARO2*) + *KmARO7*^*fbr*^ and phenylalanine (*KmARO4*^*fbr*^ + *KmARO1* + *KmARO2* + *KmARO7*^*fbr*^ + *KmPHA2* + *KmARO9*) pathways increased their production and converted more shikimate to amino acids. Chromosomal integration of the pathway further improved phenylalanine and tyrosine production. Strains marked with an asterisk (*) show a significantly higher level of 2-PE relative to that produced by ASR.004 as determined by an independent *t-*test (*p* < 0.05). The data in the bar plots are the mean ± s.d. of at least three biological replicates. All data plotted in this and following graphs can be found in Additional file 2: Supplementary Data 1

We began our engineering strategy by alleviating feedback inhibition of AAA synthesis in *K. marxianus*, a key step in establishing phenylalanine or tyrosine overproduction in yeast [9]. The *K. marxianus* enzymes (KmAro4p and KmAro7p) share > 75% sequence identity with their *S. cerevisiae* orthologues (Additional file 1: Table S1), thus we used knowledge of *S. cerevisiae* feedback resistant mutations as the basis of generating feedback resistant variants in *K. marxianus* enzymes. The substitutions K221L in KmAro4p^fbr^ and G141S in KmAro7p^fbr^ are equivalent to K229L and G141S mutations in the same enzymes in *S. cerevisiae* [9]. We initially overexpressed single copies of *KmARO4*^*fbr*^ and *KmARO7*^*fbr*^ together in *K. marxianus* strain NBRC1777 on the centromeric plasmid pA1f/A4f-U. This strain, KmASR.004, produced detectable amounts of 2-PE and tyrosol (the Ehrlich alcohol derived from tyrosine), in the culture medium as a result of AAA overflow from the feedback resistant enzymes, along with significant amounts of shikimate. Approximately 1.2—1.4 mM, or 170 mg L^−1^ of each aromatic alcohol was produced after 48 h shake-flask culture in minimal medium at 30 °C along with 1.6 mM shikimate, whereas the wild-type yeast did not produce any of these metabolites when grown for the same length of time (Fig. 2). This established that feedback resistant variants can be used in *K. marxianus* to alleviate feedback inhibition by both tyrosine and phenylalanine.

The next step was to create the centromeric plasmid pChor/A4f overexpressing both feedback-resistant alleles (*KmARO4*^*fbr*^ and *KmARO7*^*fbr*^) and two other genes of the shikimate pathway, *KmARO1* and *KmARO2.* The resulting strain, KmASR.009, did not show a significant increase in aromatic metabolite production relative to KmASR.004 (Fig. 2). Reasoning that this might be due to lack of metabolic pull downstream of Aro7p^fb*r*^, we further added two more genes, *KmPHA2* and *KmARO9,* in a new single plasmid pAAA/Phe. Pha2p pushes flux coming from chorismate to phenylalanine, and Aro9p favours formation of phenylpyruvate (PPY) over phenylalanine, thereby facilitating our approach of using 2-PE as a readout for flux through this pathway (Fig. 1). Strain KmASR.010 with these additions increased 2-PE titres by 40% relative to KmASR.009, ultimately comprising nearly 70% of the measured Ehrlich metabolites (Fig. 2; Additional file 2: Supplementary Data 1). The increased pull of the remaining enzymes downstream of Aro7p also led to less shikimate being secreted, with the acid only comprising 20% of the total aromatic products as opposed to nearly 40% for ASR.004. At this stage, it appeared that all excess phenylalanine or tyrosine was being converted to 2-PE or tyrosol, respectively; the amino acids were neither detected as extracellular nor intracellular metabolites. We also did not detect any intracellular Ehrlich metabolites, suggesting that they were all secreted during growth.

We then decided to chromosomally integrate and overexpress the same pathway in a strain lacking the native alleles of *ARO3, ARO4* and *ARO7*. Besides more stable expression, such a background would ensure that *K. marxianus* relies solely on the feedback-resistant phenylalanine/tyrosine pathway. Using CRISPR/Cas9, we inactivated *KmARO4* and *KmARO7* and then engineered a non-homologous-end-joining (NHEJ)-deficient background by inactivating the DNA ligase IV *KmDNL4* by CRISPR/Cas9 [26, 27]. The use of NHEJ mutant backgrounds was necessary to ensure that only single copies of our constructs were integrated, as this was essential to accurately evaluate engineering strategies involving gene overexpression. Furthermore, targeted chromosomal integration is more successful in NHEJ mutants. We reconstructed the Phe^fbr^ pathway in an integrative vector pI6-AAA/Phe and integrated it at the *KmARO3* locus to create strain KmASR.046. This strain lacks native Aro3p, Aro4p and Aro7p and is therefore fully reliant on the introduced enzymes for all AAA biosynthetic requirements. The combination of stable chromosomal integration of the pathway and loss of feedback-inhibited genes resulted in a final increase of nearly 40% in 2-PE production in the resultant strain KmASR.046 compared to plasmid-based expression in KmASR.010, with a final titre of 400 mg L^−1^ 2-PE and 241 mg L^−1^ tyrosol (3.31 mM and 1.75 mM, Fig. 2).

### Increasing precursor supply to the shikimate pathway

#### Increasing phosphoenolpyruvate supply to the shikimate pathway

**Fig. 3:**
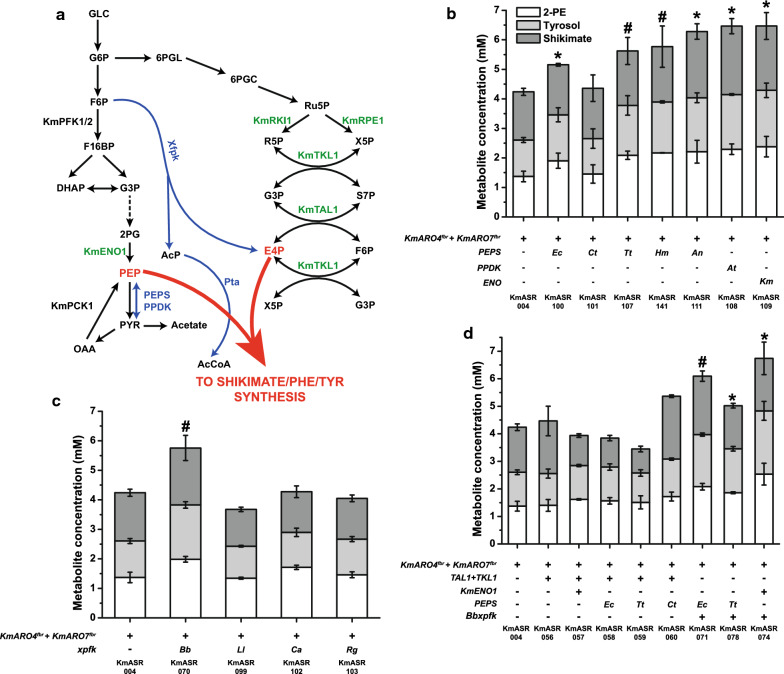
Engineering precursor supply to the shikimate pathway. **a** Overall view of the metabolic pathways giving rise to phosphoenolpyruvate (PEP) and erythrose-4-phosphate (E4P), and also the reactions that consume them. Reactions and genes in blue are heterologous, enzymes in green have their genes overexpressed, and other genes of interest are named. **b** Increasing PEP supply to the shikimate pathway heterologous using PEP synthases and pyruvate orthophosphate dikinases, and a native enolase. Enzymes of all three classes significantly increased levels of 2-PE, our proxy for phenylalanine production. **c** Increasing E4P supply to the shikimate pathway using fructose-6-phosphate phosphoketolases (Xfpk) from bacterial and fungal sources. **d** Attempts to simultaneously increase PEP and E4P supply to the shikimate pathway showed that the effects of increasing either metabolite individually are not additive, though in the end a modification was found that increased 2-PE production beyond what was achieved by individual precursors. Strains producing significantly higher amounts of 2-PE relative to that produced by ASR.004 as determined by an independent *t-*test are marked by an asterisk (*p < 0.05) or a hash (^#^*p* < 0.005). All data are plotted as the mean ± s.d. of at least three biological replicates. *Ec*: *Escherichia coli*, *Ct*: *Chaetomium thermophilum*, *Tt*: *Thielavia terrestris, Hm: Halofax mediterranei, An: Aspergillus nomius, At: Arabidopsis thaliana, Km: Kluyveromyces marxianus, Bb: Bifidobacter breve, Ll: Lactococcus lactis, Ca: Clostridium acetobutylicum*, *Rg: Rhodotorula graminis*

We next decided to investigate the best means to increase phosphoenolpyruvate (PEP) and erythrose-4-phosphate (E4P) supply to the shikimate pathway. PEP is a substrate in two steps of the shikimate pathway, required by Aro4p and Aro1p (Fig. 1). In yeast, PEP is produced by enolase (Eno1/2p) during glycolysis and PEP carboxykinase (Pck1p) during gluconeogenesis (Fig. 3a). In other species, PEP is also synthesised by PEP synthases (PEPS, EC 2.7.9.2) or pyruvate orthophosphate dikinase (PPDK, EC 2.7.9.1), enzymes that reversibly produce PEP from pyruvic acid [28]. In *E.coli,* the PEPS gene *EcppsA* has been frequently used to increase precursor supply to produce aromatic amino acid-derived compounds [29, 30]. Although *EcppsA* was unsuccessfully tested for a similar role in one *K. marxianus* strain [23], we decided to test it and several of these enzymes*,* initially in KmASR.004 and eventually in KmASR.046 with the full Phe^fbr^ (all steps from E4P/PEP to Phe) background*.* We overexpressed five codon-optimised PEPSes: *EcppsA*, an archaeal PEPS and three fungal PEPSes (Table 1). The fungal PEPSes were chosen based on their sequence homology to *EcppsA,* while the archaeal PEPS was chosen since it was reported to solely catalyse PEP formation [31]. We also chose to test a codon-optimised PPDK from *Arabidopsis thaliana* as it has a tenfold higher* K*_m_ for pyruvate than PEP as substrate, which could allow for efficient conversion of pyruvate to PEP [28]. We also included the native *ENO1* from *K. marxianus*. Each of these genes was individually introduced into KmASR.004 and the effect on 2-PE production assessed (Fig. 3b). All three classes of enzyme were able to increase aromatic amino acid and shikimate production by providing more PEP for AAA synthesis. Among the heterologous enzymes, the PEPS from *Aspergillus nomius* (ASR.111) and PPDK from *A. thaliana* (ASR.108) were the best performing, both increasing 2-PE production to over 2 mM (Fig. 3b). Overexpressing *KmENO1* proved to be the most effective strategy to increase PEP, producing nearly 2.4 mM 2-PE, which represented an increase by over 70% relative to ASR.004 and was nearly identical to the titre produced using the entire Phe^fbr^ pathway (KmASR.010 vs KmASR.109, Fig. 3b).

**Table 1:** Gene name, source organism and GenBank ID for heterologous genes used in this study. Nucleotide sequences are provided in Additional file 3: Supplementary Data 2

Gene/protein ID	Source organism	GenBank accession no
*EcppsA/EcPEPS*	*Escherichia coli*	P23538.5
*CtPEPS*	*Chaetomium thermophilum*	EGS22573.1
*TtPEPS*	*Thielavia terrestris*	AEO67530.1
*HmPEPS*	*Haloferax meditarranei*	AFK18505.1
*AnPEPS*	*Aspergillus nomius*	KNG90458.1
*AtPPDK*	*Arabidopsis thaliana*	AEE83616.1
*Bbxfpk*	*Bifidobacterium breve*	KND53308.1
*Llxfpk*	*Lactococcus lactis*	AAK05600.1
*Caxfpk*	*Clostridium acetobutylicum*	KHD36088.1
*Rgxfpk*	*Rhodotorula graminis*	KPV77773.1
*Bspta*	*Bacillus subtilis*	CAB15793.1
*Septa/eutD*	*Salmonella enterica*	OAQ12105.1

#### Increasing erythrose-4-phosphate supply to the shikimate pathway

The natural source for E4P is the non-oxidative branch of the pentose phosphate pathway (PPP) via cross-reactions from transketolase (Tkl1p) or transaldolase (Tal1p) (Fig. 3a). In *S. cerevisiae,* either or both of these enzymes are typically overexpressed to increase available E4P for AAA synthesis. More generally, metabolic engineering studies in *S. cerevisiae* revealed that partial or complete overexpression of different parts of the non-oxidative PPP can increase the production of AAA or shikimate-derived products in combination with other modifications [6, 10, 11]. As *K. marxianus* has previously been reported to have a higher flux through the PPP than *S. cerevisiae* [32, 33], it was unclear whether overexpressing these enzymes would significantly improve E4P levels in *K. marxianus*. An alternative strategy tested in *S. cerevisiae* was to express a non-native fructose-6-phosphate phosphoketolase (Xfpk, EC 4.1.2.22), which converts fructose-6-phosphate to E4P and acetyl phosphate (Fig. 3a) [34]. We tested four phosphoketolases reported in the literature: three from *Bifidobacterium breve, Lactococcus lactis* and *Rhodotorula graminis* [34–36], as well as a phosphoketolase from *Clostridium acetobuytlicum* that favours xylulose-5-phosphate as a substrate over fructose-6-phosphate (Fig. 3c). Of the four phosphoketolases, only the Xfpk from *B. brevis* (*Bbxfpk*) increased the production of aromatic metabolites significantly, with 2-PE production increasing to over 2 mM (KmASR.070). However, in all strains, this was accompanied by increased acetate production (Additional file 1: Fig. S2a) as well as slower growth compared to KmASR.004. In contrast, overexpressing *KmTKL1* and *KmTAL1* together (KmASR.056, Fig. 3d) did not significantly affect production of metabolites, with a slight increase in shikimate balanced overall by a decrease in tyrosine.

Finally, we decided to examine which combinations of enzymes best increased both precursors in KmASR.004 by coexpressing *KmENO1* or different PEPSes alongside either *Bbxfpk* or the Km*TKL1/TAL1* combination. Relative to our previous attempts to increase phenylalanine production one precursor at a time, only a combination of BbXfpk and KmEno1 further incrementally improved production (KmASR.074, Fig. 3d ), but this was not significantly higher than KmASR.070 or KmASR.109. This suggested that the combined effects of enzymes used to increase PEP or E4P supply to the shikimate pathway were not purely additive. Furthermore, neither the PEPSes nor *KmENO1,* when coexpressed with *KmTKL1* and *KmTAL1* in KmASR.004, significantly increased production (KmASR.057-KmASR.059, Fig. 3d ), nor did coexpressing PEPSes alongside *Bbxfpk* increase production over KmASR.070 (KmASR.070 vs KmASR.071, Fig. 3d).

### Integrating precursor supply with the phenylalanine biosynthetic pathway

**Fig. 4:**
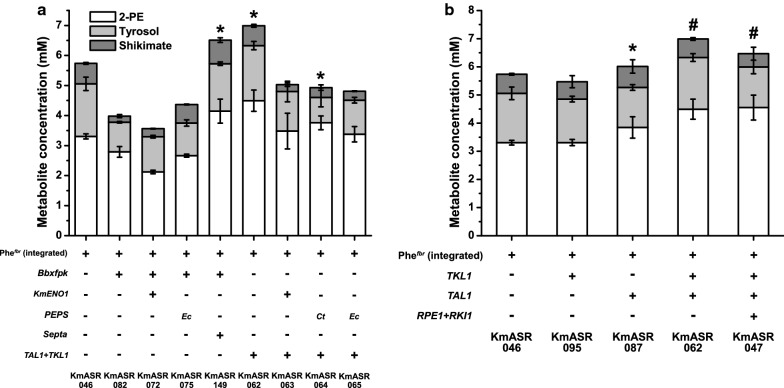
Optimising phenylalanine production by increasing precursor supply to the phenylalanine pathway. **a** Overexpressing heterologous enzymes to increase precursor supply either by themselves or with native enzymes had more variable results, with strategies that worked in KmASR.004 not working in KmASR.046. Ultimately precursor supply via the PPP (KmASR.062) could be matched by using a phosphoketolase (Xfpk) from B. breve and a phosphotransacetylase (Pta) from S. enterica (KmASR.149). **b** Using a partial (KmASR.062) or full (KmASR.047) overexpression of the non-oxidative pentose phosphate pathway (PPP), we were able to increase phenylalanine production in strain KmASR.046 overexpressing a feedback-resistant phenylalanine pathway by 33%. Strains producing significantly higher amounts of 2-PE relative to that produced by KmASR.046 as determined by an independent t-test are marked by an asterisk (*p < 0.05) or a hash (^#^*p* < 0.005) All data are plotted as the mean ± s.d. of at least three biological replicates. *Km: Kluyveromyces marxianus, Ct: Chaetomium thermophilum, Tt: Thielavia terrestris, Bb: Bifidobacter breve, Se: Salmonella enterica*

Our engineering of KmASR.004 identified strategies that could increase PEP and E4P supply to the shikimate pathway. We now wanted to test how these strategies would perform in a strain optimised for AAA production, which should exhibit downstream pull through the shikimate pathway. Unexpectedly, the two modifications that had been identified as the best performing in KmASR.004, overexpression of *Bbxfpk* (KmASR.082) by itself or with *KmENO1* (KmASR.072), performed poorly and led to a reduction in titres of 2-PE (and tyrosol and shikimate) when introduced into KmASR.046 (Fig. 4a). One possible explanation was that the acetyl phosphate produced by Xfpk is further converted to acetate, lowering the cytosolic pH and increasing metabolic burden [37]. If the observed changes in 2-PE production are taken to be a proxy for a shifting metabolic burden, this appears to be the case because the overexpression of a phosphotransacetylase (Pta, EC 2.3.1.8) from *Salmonella enterica* (*Septa*) [38] along with *Bbxfpk* alleviated the problem and generated strain KmASR.149 that produced over 4 mM 2-PE. We also tested the effects of overexpression of enzymes of the non-oxidative branch of the PPP (Fig. 4b). While individual effects of *KmTKL1* and *KmTAL1* were weak, overexpression of both *KmTKL1* and *KmTAL1* (KmASR.062) or the entire non-oxidative branch of the PPP (KmASR.047) yielded ~ 4.5 mM 2-PE. Combining overexpression of *KmTKL1* and *KmTAL1* (KmASR.062) with *KmENO1* (KmASR.063) or bacterial PEPS (KmASR.064, KmASR.065) did not improve the titres of 2-PE (Fig. 4a), again illustrating that modifications are not simply additive.

**Fig. 5:**
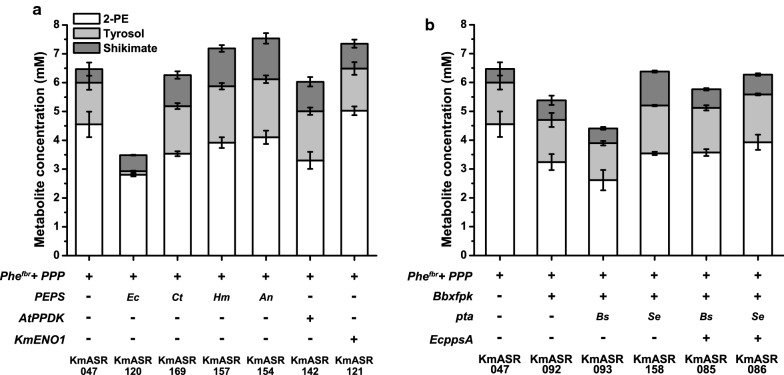
Further integration of precursor supply in a phenylalanine overproducing strain. **a** Beginning with strain KmASR.047 with the non-oxidative PPP overexpressed, we attempted to further increase PEP supply using *EcppsA*, the PEP synthase from *E.coli*, a PPDK from *A. thaliana* and the *K. marxianus* enolase, with only the last increasing phenyalanine/-2-PE production. **b** Attempts to further increase E4P supply using Xfpk resulted in net decreases in phenylalanine, tyrosine and shikimate production. Expressing a Pta only allowed aromatic compound production to recover if an enzyme was coexpressed to hypothetically simultaneously increase PEP supply. We also found that the Pta from *S. enterica* was more effective in this role. Data are the mean ± s.d. of at least three biological replicates. *Km: Kluyveromyces marxianus, At: Arabidopsis thaliana, Bb: Bifidobacterium breve, Ct: Chaetomium thermophilum, An: Aspergillus nomius, Hm: Haloferax mediterranei, Bs: Bacillus subtilis, Se: Salmonella enterica*

Comparing all the derivatives of KmASR.046 tested, KmASR.047, KmASR.062 and KmASR.149 had the most improved production of phenylalanine, all producing up to 4.5 mM (550 mg L^−1^) 2-PE. Of these three, we decided to use KmASR.047 as the basis for integrating strategies to simultaneously increase E4P and PEP supply to phenylalanine biosynthesis. While the three mentioned strains produced approximately similar levels of 2-PE, tyrosol and shikimate, KmASR.047 began producing larger amounts of all aromatic metabolites within 24 h (Additional file 1: Fig. S3), compared to the other two strains, suggesting that this strain could be better optimised for faster bioprocesses to reach desired titres within a shorter fermentation time. However, the growth of the strains did not significantly differ, all having doubling times between 148 and 162 min when grown on minimal medium with glucose. We now took KmASR.047 as a base strain and considered approaches to improve PEP supply using the knowledge that we had gained. Because we had already found that interactions between pathways were complex, we also retested some of the enzymes that had not performed well previously (Fig. 5a). *KmENO1* increased 2-PE production (KmASR.121), whereas overexpressing *EcppsA* or PPDK led to a significant decrease in the production of 2-PE (KmASR.120 and KmASR.142; Fig. 5a). Using the PEPSes from *Aspergillus nomius* (*AnPEPS*; KmASR.154) or *Haloferax mediterranei* (*HmPEPS*; KmASR.157) also reduced 2-PE titres but increased shikimate concentrations to ~ 2 mM, compared to 0.46 mM in KmASR.047, with a net increase in total aromatic metabolites (Fig. 5a). It may be that these enzymes, catalysing PEP formation reversibly, began to synthesise pyruvate from PEP and deplete levels of the latter in the metabolic background of KmASR.047, leading to insufficient PEP to carry out the Aro1p-catalysed reaction (Fig. 1).

Further attempts to increase E4P supply in KmASR.047 using phosphoketolase were also unsuccessful (Fig. 5b). Overexpressing *Bbxfpk* in this strain decreased 2-PE production by nearly 30% to 3.24 mM (KmASR.092) and, in contrast to the previous finding with KmASR.046, this effect was not alleviated by coexpressing *Septa* (KmASR.158). We also tested an additional PTA, *Bspta* from *B. subtilis,* but this also did not recover production (KmASR.093). An excess of E4P is known to inhibit bacterial DAHP synthase in the absence of PEP [39] and could be relieved by addition of excess PEP. We therefore coexpressed *EcppsA* alongside *Bbxfpk* and the two Ptas creating strains KmASR.085 and KmASR.086. While this did improve production over KmASR.093 and KmASR.0158, increasing production overall and converting more shikimate to 2-PE and tyrosol, 2-PE production was still less than KmASR.047. Taken together, these data suggest that careful balancing of the precursors to the phenylalanine biosynthetic pathway must be taken into consideration when increasing their availability.

### Optimisation of 2-phenylethanol production by knock-down of tyrosine production and aromatic aminotransferase deletion

**Fig. 6:**
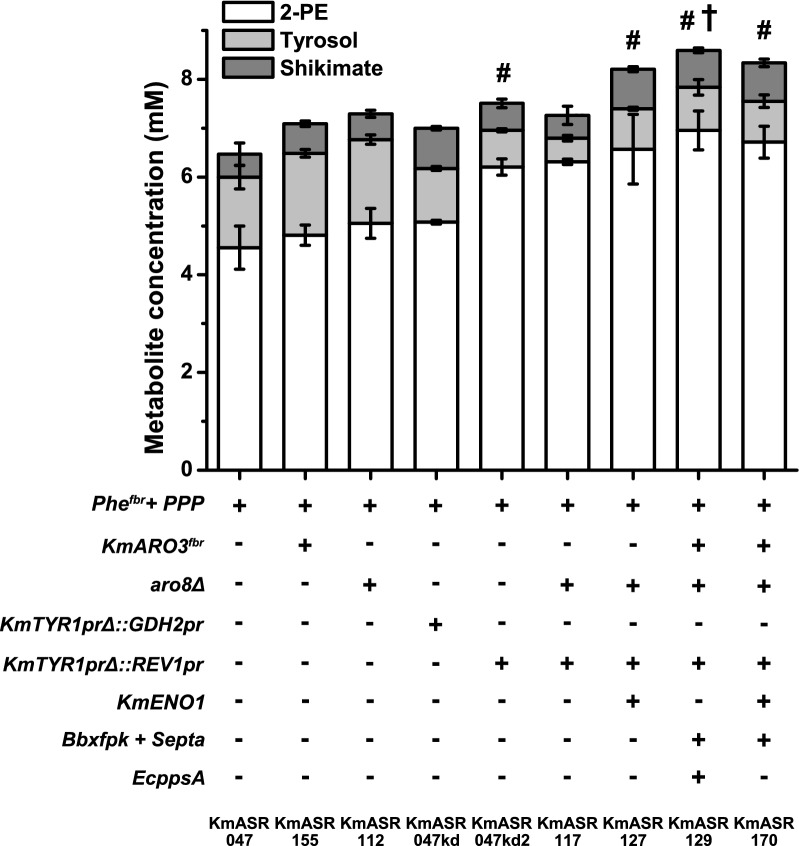
Optimising phenylalanine production by minimising degradation and competition from tyrosine biosynthesis. KmASR.047′s aromatic amino acid biosynthetic flux was redirected by knocking down *TYR1* expression using a weak promoter (ASR.047kd, ASR.047kd2), and restricting metabolism by knocking out the aromatic aminotransferase *KmARO8* (KmASR.112). The most effective promoter knockdown, the *REV1* promoter, was combined in the latter to produce KmASR.117. With the increased capacity for phenylalanine synthesis in this strain, we increased phenylalanine synthesis more by further supplying precursors using either *KmENO1* or a combination of heterologous enzymes alongside *KmARO*3^*fbr*^ to better utilise increased precursor levels. Data are the mean ± s.d. of at least three biological replicates. trains producing significantly higher amounts of 2-PE relative to that produced by KmASR.047 as determined by an independent *t-*test are marked by an asterisk (*p < 0.05) or a hash (^#^*p* < 0.005), and those producing significantly more 2-PE relative to KmASR.0117 are marked with a dagger (†*p* < 0.05). Data are the mean ± s.d. of at least three biological replicates. *Km: Kluyveromyces marxianus, Bb: Bifidobacterium breve, Se: Salmonella enterica*

The 2-PE produced by the best-performing strains so far only amounts to approximately 70% of the total aromatic metabolites produced and 74% of the Ehrlich metabolites of phenylalanine and tyrosine. The third step of our engineering strategy is to knock down expression of the prephenate dehydrogenase *KmTYR1* (Fig. 1). This enzyme converts prephenate to 4-hydroxphenylpyruvate, committing it to tyrosine synthesis: by reducing its expression as low as possible without creating a tyrosine auxotroph, more prephenate from Aro7p should be available for conversion to phenylpyruvate and phenylalanine. To achieve this, we substituted the native *KmTYR1* promoter (*TYR1pr*) with one of two weaker promoters, *GDH2pr* and *REV1pr,* that we had previously characterised [27]. Both strains, KmASR.047kd and KmASR.047.kd2, increased the relative proportion of 2-PE to tyrosol, with the strongest effect seen when *REV1pr* (strain KmASR.047kd2) was used (Fig. 6). At this stage, 2-PE amounted to 89% of the Ehrlich metabolites and 83% of the aromatic metabolites measured. The decreased tyrosine production was still sufficient for prototrophy, and neither of the knock-downs created a significant growth defect in KmASR.047. Another strategy reported in the literature to increase 2-PE production is to delete the aromatic aminotransferase encoded by *ARO8* [40]. Inactivation of *KmARO8* using CRISPR/Cas9 caused a 10% increase in all measured metabolites, with 2-PE titres increasing to 5.05 mM (KmASR.112). We subsequently combined the two strategies in KmASR.047 to examine if these effects were additive for the production of 2-PE (KmASR.117). In KmASR.117, 2-PE production did not significantly increase (6.31 mM vs 6.25 mM in KmASR.047kd2) but formed nearly 93% of the total Ehrlich metabolites, as opposed to 89% and 82%, respectively for the *KmTYR1* knockdown and *KmARO8* knockout alone (Fig. 6). The same modifications, when made to KmASR.062, resulted in a smaller increase in aromatic compound production resulting in the production of 5.2 mM 2-PE (~ 634 mg L^−1^; Additional file 1: Fig. S4).

### Creation of a high 2-phenylethanol – producing strain by a synthesis of tested engineering strategies

The three parts of our engineering strategy so far allowed us to establish feedback-resistant phenylalanine synthesis, optimise E4P supply for phenylalanine synthesis and then redirect metabolic flux within this pathway to create strain KmASR.117. However, lessons learned from other strategies to increase PEP and E4P supply led us to attempt to also increase precursor supply in this strain. Overexpressing *KmENO1* in KmASR.117, led to a further increase in all aromatic compounds produced overall but with 2-PE only increasing by 4% (KmASR.127, Fig. 6). We then decided to take what were now our best strains and see what the effect of modifications for increase of E4P and/or PEP would be. We also included a feedback resistant allele of *KmARO3* since an *ARO3*^*fbr*^ created in *S. cerevisiae* was found to aid aromatic amino acid synthesis when used in tandem with *ARO4*^*fbr*^ [41]*.* Based on the performance of previous strains, we hypothesised that KmAro3p^fbr^ would allow faster consumption of the increased E4P and PEP, avoiding their accumulation and diversion to other reactions. *KmARO3*^*fbr*^ encoding a K222L mutation was overexpressed in KmASR.117 alongside *Bbxfpk, Septa* and *EcppsA* or *KmENO1* to create strains KmASR.129 and KmASR.170, respectively. Of the two, the best performing strain was KmASR.129, which produced nearly 850 mg L^−1^ (6.95 mM) 2-PE in 48 h, the highest titre so far, with a yield of 0.062 mol 2-PE/mol of glucose. Together, the final yield of aromatic metabolites (2-PE, tyrosol, shikimic acid) from KmASR.129 was 0.077 mol/mol of glucose.

## Discussion

*K. marxianus* is a promising next-generation yeast cell factory on account of its unique physiology, and has begun to be used for such applications. The same physiology and differences in metabolism to *S. cerevisiae* means that metabolic engineering approaches for this yeast require novel, rational approaches to best redirect its metabolism to generate precursors for valuable chemical compounds. We implemented a three-step rational strategy to optimise a native biosynthetic pathway for overproduction and demonstrated its use for production of a commercially-relevant product de novo from synthetic growth medium. By (i) establishing phenylalanine overexpression via a feedback-resistant biosynthetic pathway, (ii) increasing precursor supply to the pathway, and (iii) knocking down competing pathways, reducing degradation and finally re-balancing precursor supply, our best-producing strains could synthesise 6.95 mM, or nearly 850 mg L^−1^, of 2-phenylethanol (2-PE) from glucose in 48 h flask cultivation. While we engineered *K. marxianus* for the production of 2-PE, relatively few modifications would be required to convert this strain to a phenylalanine production strain. The main modification would involve the inactivation of *KmARO10* and *KmPDC5* to ensure the conversion of phenylpyruvate (PPY) to phenylalanine as opposed to 2-PE. This strain would be a platform for production of phenylalanine-derived products.

Keeping *K. marxianus’* unique physiology in mind, we overexpressed heterologous enzymes that would make use of its high TCA cycle and PPP fluxes to increase precursor supply to the shikimate pathway [32, 33, 42]. By screening several PEP synthases, we were able to identify at least two, AnPEPS (in strains KmASR.108 and KmASR.154), and EcPEPS (in KmASR.129), that effectively convert pyruvate to PEP for the shikimate pathway in strains overexpressing a feedback resistant (fbr) phenylalanine biosynthetic pathway. Although a previous study suggested that EcPEPS could not function in this manner in *K. marxianus* [23], we found that the choice of PEPS and strain engineering played a role in the extent to which PEP synthase overexpression improved AAA or shikimate production. As some PEPSes are exclusively used by their host organisms for either gluconeogenesis or PEP synthesis [31, 43], a further investigation of more PEPSes with similar functions and kinetics may help us identify the best way to integrate this class of enzyme in yeast cell factories. In our efforts to increase E4P production, we overexpressed phosphoketolases that tap into *K. marxianus’* high fructose-6-phosphate levels and PPP flux [33, 37] to further boost available E4P. This strategy has recently been employed in other yeasts to increase the production of phenylalanine and tyrosine-derived metabolites with mixed results [12, 44–46]. While it was successful in *K. marxianus*, we found that an Xfpk’s ability to increase the production of aromatic compounds is tempered by the extent of metabolic engineering on the AAA pathway. In contrast to previous studies in *S. cerevisiae* [45], we found that coexpressing a phosphotransacetylase (Pta) with *Bbxfpk* did improve production relative to when just *Bbxfpk* was expressed but that the choice of Pta mattered (KmASR.149, Fig. 4a; KmASR.158, Fig. 5b). Because as in *S. cerevisiae*, the overexpression of an Xfpk led to increased acetate secretion (Additional file 1: Fig. S2) [44], we suppose that expression of the Pta relieves metabolic burden. Further metabolomic studies on the impact of these combinations of enzymes on *K. marxianus*’ metabolism, especially the TCA cycle and acetate metabolism, would be required to fully assess the metabolic burden that causes a decrease in 2-PE production (KmASR.082 vs KmASR.046, Fig. 4a; KmASR.092 vs KmASR.047, Fig. 5b).

Our final strain modifications highlighted the importance of balancing precursor supply and ensuring that precursors are efficiently utilised. This was especially apparent when optimal strategies to increase E4P and PEP varied across different scales of feedback-resistant 2-PE biosynthesis (Fig. 3, Fig. 5). Certain engineering strategies remain to be tried, such as mutating or deleting pyruvate kinase [12, 46], or utilising gluconeogenic enzymes to increase PEP. However, a combinatorial fine-tuning of gene expression, especially for PEP and E4P-supplying enzymes, could provide a more robust and scalable strategies to increase precursor supply since other modifications, such as the overexpression of *ARO*3^*fbr*^, only had an effect on 2-PE production when precursor supply was sufficiently increased for the enzyme to optimally function. Finally, as our best performing strains still leaked some shikimic acid from the pathway, overexpression of a bacterial shikimate kinase in addition to *KmARO1* could be considered to ensure all the shikimate synthesised is used downstream and not secreted [12, 47]. In a broader sense, native enzymes in the shikimate and phenylalanine pathways could further be replaced by heterologous, more kinetically efficient homologues, or more multifunctional enzymes with equivalent activity [41].

One significant difference to *S. cerevisiae* engineering approaches we found was that whereas in *S. cerevisiae* overexpressing transketolase alone is sufficient to increase E4P levels, in *K. marxianus*, overexpression of both transketolase and transaldolase is required. While besides having a naturally high flux through the PPP, *K. marxianus* has also been reported to have high intracellular levels of fructose-6-phosphate [33], one of the substrates for transketolase but for the reverse reaction of interest; these high levels may inhibit the production of E4P by the overexpressed *KmTKL1* and drive equilibrium in the opposite direction. By coexpressing it with *KmTAL1*, the two enzymes may be acting in tandem through their reactions, to ensure E4P production and avoiding the drawbacks of a single enzyme running in the reverse direction. Beyond this, overexpressing the rest of the pathway did not significantly improve phenylalanine production but provided a number of advantages such as a faster onset of production and more capacity for production once competing pathways were eliminated (Additional file 1: Figs. S3, S4).

The focus of our study was on developing a rational synthetic biology framework for building a *K. marxianus* chassis to overproduce aromatics and demonstrating its potential for strain engineering. With the caveat that different experimental set-ups make exact comparisons difficult, the titre (6.95 mM) and yield (62 mmol / mol glucose) of 2-PE achieved by our best-performing strain (KmASR.129) are within the range reported for other engineered de novo 2-PE producing yeasts: *P. pastoris* (87 mmol/mol); *Y. lipolytica* (97 mmol/mol); and *S. cerevisiae* (113 mmol/mol) [12, 46, 48]. Interestingly, derivatives of a different *K. marxianus* strain (DMKU3-1042) were reported to produce up to 1.3 g 2-PE L^−1^ (10.7 mM) [23]. That study used a combination of rational engineering and laboratory evolution, which is a strategy that is routinely followed when moving from a prototype towards a production strain and process. Since we were interested in developing a platform rather than a bioprocess, we did not pursue this route, but it seems likely that the full potential of our *K. marxianus* strain for 2-PE production has not yet been reached. There are some notable differences between our findings with strain NBRC1777, and that study with strain DMKU3-1042, which illustrate the variation that strain selection can bring. Kim and colleagues report that overexpression of *ARO10* and *ADH2* from *S. cerevisiae* led to a significant increase in 2-PE production. In contrast, in our strain background, overexpressing the same genes in wild-type, KmASR.047 or KmASR.117 had only marginal effects (Additional file 1: Fig. S5). Based on these data and other indicators, it is likely that underlying differences in AAA metabolism between the two strains influence the effectiveness of particular engineering approaches, and more knowledge in this area is required. Future research into the comparative metabolomics of *K. marxianus* strains, especially with regard to amino acid and nitrogen metabolism, will help identify the most metabolically suited strains for specific applications.

## Conclusion

In summary, we implemented a comprehensive synthetic biology strategy to build a *K. marxianus* strain for production of molecules derived from the aromatic amino acid phenylalanine. The potential of this chassis was demonstrated by constructing a cell factory that, starting from zero, produced almost 7 mM 2-PE from minimal mineral medium and glucose. Relatively few further modifications would be required to redirect metabolism for alternative products, or indeed to use tyrosine or tryptophan as the starting point. It would also be very interesting to apply our synthetic approach to the use of alternative carbon sources, in particular second generation feedstocks. Our study combined native and heterologous enzymes to increase the supplies of both phosphoenolpyruvate and erythrose-4-phosphate, and while not exhaustive, our findings reveal that balancing the precursors to shikimate and phenylalanine biosynthesis not only affects the amount but also the profile of aromatic metabolites produced. In two key aspects, the importance of synthetic biology approaches were very evident. First, it was necessary to try multiple versions of the same enzyme to identify the most suitable one for a particular reaction; and second, the effectiveness of any given modification was influenced in non-predictable ways by other modifications. Thus, a combinatorial, parallel approach is required to build high-performing strains. Although such strategies have been deployed with *S. cerevisiae* for some time, it was considered that the development of other yeasts was not sufficiently advanced to allow the same approach. With this study, we demonstrate that *K. marxianus* has reached the stage where synthetic biology is feasible and the steps that we followed may also serve as a roadmap for other non-traditional, or non-conventional, yeast cell factories.

## Materials and methods

### Strains and media

*Kluyveromyces marxianus* strain NBRC1777 from the Biological Resource Centre, NITE (NBRC; Tokyo Japan) was used as the base strain for all work in this study. For preliminary genome editing and knock-outs, strains were routinely grown in YPD broth (2% yeast extract, 1% peptone and 2% glucose; Formedium, Hunstanton, UK), or synthetic drop-out (SD) medium with the relevant supplements (0.5% ammonium sulphate, 0.19% yeast nitrogen base without amino acids or ammonia (Formedium), 2% glucose and the relevant drop-out supplement according to the manufacturer’s recommendations (Formedium)). When cells needed to be grown on solid media, the recipes above were supplemented with 2% agar (Formedium). For transformations involving CRISPR/Cas9, strains were grown on YPD agar with 200 mg L^−1^ hygromycin for selection. Bacterial transformations used *E.coli* DH5a grown in LB medium (1% NaCl, 1% peptone, 0.5% yeast extract; Formedium) or LB agar (+ 1.5% agar) with the appropriate antibiotic (100 mg L^−1^ ampicillin, 50 mg L^−1^ chloramphenicol or 50 mg L^−1^ kanamycin) as required. All antibiotics and reagents were purchased from Fisher Scientific (Dublin, Ireland) or Sigma-Alrdich/Merck (Haverhill, UK). All primers were obtained from Sigma-Aldrich.

### Plasmid construction

All genes for the AAA biosynthetic pathway and non-oxidative PPP were amplified from genomic DNA from *K. marxianus* strain CBS6556 (Westerdijk Fungal Biodiversity Institute, Utrecht, The Netherlands) using Q5 High-Fidelity Polymerase (M4092L, New England Biolabs (NEB), Ipswich, UK) with flanking nested BsaI (R0535L, NEB) and BsmBI (R0739L, NEB) restriction enzyme sites added by PCR. All synthetic heterologous genes were codon-optimised for *S. cerevisiae* and obtained from Twist Biosciences (San Francisco, U.S.A.). Sequences for these genes are available in Additional file 3: Supplementary Data 2. These respectively were used to clone the genes into storage plasmids (level I) by Golden Gate assembly with BsmBI and T7 DNA ligase (M0318L, NEB) for subsequent assembly into expression cassette-bearing plasmids (level II) using the hierarchical system based on the Yeast Toolkit standard [49]. Component vectors containing markers and connectors used to construct cloning vectors using this standard were a gift from John Dueber distributed through (Kit # 1000000061, Addgene, Watertown, U.S.A.). The genes or gene fragments were cloned into the storage vector pYTK001 using a Golden Gate assembly with BsmBI. This plasmid contains a GFP cassette in its ‘empty’ state, allowing for green/white colony screening. Each plasmid’s insert was verified by colony PCR following transformation into *E.coli,* and subsequent sequencing of the inserts of PCR-positive plasmids. For constructing feedback-resistant alleles of *KmARO4* and *KmARO7*, the mutations were made to the genes during Golden Gate assembly by amplifying the gene as two fragments flanking the residue to be mutated. The mutated residue (K221L in *KmARO4* and G141S in *KmARO7*) was included in the complementary overhangs for the BsmBI enzyme sites meant to join the fragments, so that on successful assembly of the fragments into a level I plasmid, the cloned product is the entire gene with the incorporated mutation. A similar strategy was used to eliminate internal BsaI or BsmBI sites that would otherwise interfere with Golden Gate assembly.

Level I plasmids for each gene and the desired promoter and terminator were then assembled into transcriptional units (TUs) by Golden Gate assembly with BsaI/T7 DNA ligase, which were then transformed into *E.coli* and verified by colony PCR using OneTaq Quick-Load Polymerase (M0486L, NEB) and restriction digestion by NotI (FD0596, Fisher Scientific). The *K. marxianus* promoters and terminators used for these plasmids have been described elsewhere [27]. Depending on their intended positions in a multi-TU plasmid, TUs in level II plasmids were cloned with direction-specific connectors flanked by BsmBI sites provided in the Yeast Toolkit to ensure directional assembly. Depending on the planned constructs, some TUs were cloned multiple times with different connectors. Finally, multiple TUs comprising pathways or parts of pathways were cloned into multi-TU plasmids by a Golden Gate assembly of the TU plasmids into drop-out expression vectors constructed in-house using BsmBI/T7 DNA ligase. All the pathway expression plasmids and cloning vectors are listed in Table 2. All level I and level II plasmids constructed for this study are listed in Additional file 1: Table S2, and the primers used to create them in Additional file 1: Table S3. More detailed information on Golden Gate assembly of the plasmids can be found in refs [27, 49].

**Table 2:** Expression plasmids used in this study. Unless specified otherwise, all genes, promoters and terminators in the inserts are from *K. marxianus*

Name	Contents/comments	Source
pUCC001	*AaTEF1pr-SpCas9-ScPHO5t ScTDH3pr-HH-gRNA*_*empty*_*-HDV-ScCYC1t hphMX*	[51]
pMTU-DO-URA	Centromeric plasmid with GFP drop-out; *ScURA3 KmARS/CEN7*; available from Addgene (#160004)	This work
pMTU-DO-HIS	Centromeric plasmid with GFP drop-out; *ScHIS3 KmARS/CEN7*; available from Addgene (#160005)	This work
pI1-MTU-DO-HIS	Integrative plasmid with GFP drop-out targeting *LAC4*; *ScHIS3*; available from Addgene (#160142)	This work
pI6-MTU-DO-URA	Integrative plasmid with GFP drop-out targeting *ARO3*; *ScURA3*; available from Addgene (#160030)	This work
pI2-MTU-DO-G418	Integrative plasmid with GFP drop-out, *kanMX*, targeting integration site I2 [27]; available from Addgene (#160018)	This work
pI4-MTU-DO-G418	Integrative plasmid with GFP drop-out, *kanMX,* targeting integration site I4 (Rajkumar et al., 2019); available from Addgene (#160215)	This work
pA1f/A4f-U	pMTU-DO-URA with insert *PDC1pr-ARO4*^*fbr*^*-ScADH1pr TEF1pr-ARO7*^*fbr*^*-INU1t*	This work
pChor/A4f	pMTU-DO-URA with insert *PDC1pr-ARO4*^*fbr*^*-ScADH1pr TSA1pr-ARO1-KMXK_A03020t PGK1pr-ARO2-LAC4t TEF1pr-ARO7*^*fbr*^*-INU1t*	This work
pAAA/Phe	pMTU-DO-URA with insert *PDC1pr-ARO4*^*fbr*^*-ScADH1pr TSA1pr-ARO1-KMXK_A03020t PGK1pr-ARO2-LAC4t TEF1pr-ARO7*^*fbr*^*-PDC1t ENO1pr-PHA2-INU1t HSP150pr-ARO9-ScPGK1t*	This work
pI6-AAA/Phe	pI6-MTU-DO-URA with insert *PDC1pr-ARO4*^*fbr*^*-ScADH1pr TSA1pr-ARO1-KMXK_A03020t PGK1pr-ARO2-LAC4t TEF1pr-ARO7fbr-PDC1t ENO1pr-PHA2-INU1t HSP150pr-ARO9-ScPGK1t*	This work
pI1-P8A9T1	pI1-MTU-DO-HIS with insert *TEF1pr-TKL1-INU1t*	This work
pI1-P1A11T5	pI1-MTU-DO-HIS with insert *PGK1pr-TAL1- KMXK_A03020t*	This work
pI1-PPP	pI1-MTU-DO-HIS with insert *TEF1pr-TKL1-INU1t PGK1pr-TAL1- KMXK_A03020t TDH3pr-RPE1-PDC1t TSA1pr-RKI1-ScPGK1t*	This work
pI1-TKAL	pI1-MTU-DO-HIS with insert *TEF1pr-TKL1-INU1t PGK1pr-TAL1- KMXK_A03020t*	This work
pI1-TKAL-ENO	pI1-MTU-DO-HIS with insert *TEF1pr-TKL1-INU1t PGK1pr-TAL1- KMXK_A03020t SSA2pr-KmENO1-ScADH1t*	This work
pI1-TKAL-A26	pI1-MTU-DO-HIS with insert *TEF1pr-TKL1-INU1t PGK1pr-TAL1- KMXK_A03020t SSA2pr-CtPEPS-ScADH1t*	This work
pI1-TKAL-A27	pI1-MTU-DO-HIS with insert *TEF1pr-TKL1-INU1t PGK1pr-TAL1- KMXK_A03020t SSA2pr-EcppsA-ScADH1t*	This work
pPPP-ENO	pMTU-DO-HIS with insert *TEF1pr-TKL1-INU1t PGK1pr-TAL1- KMXK_A03020t SSA2pr-KmENO1-ScADH1t*	This work
pPPP-A26	pMTU-DO-HIS with insert *TEF1pr-TKL1-INU1t PGK1pr-TAL1- KMXK_A03020t SSA2pr-CtPEPS-ScADH1t*	This work
pPPP-A27	pMTU-DO-HIS with insert *TEF1pr-TKL1-INU1t PGK1pr-TAL1- KMXK_A03020t SSA2pr-EcppsA-ScADH1t*	This work
pPPP-A28	pMTU-DO-HIS with insert *TEF1pr-TKL1-INU1t PGK1pr-TAL1- KMXK_A03020t SSA2pr-TtPEPS-ScADH1t*	This work
pTKAL	pMTU-DO-HIS with insert *TEF1pr-TKL1-INU1t PGK1pr-TAL1- KMXK_A03020t*	This work
pP6B14T6-HIS	pMTU-DO-HIS with insert *HSP150pr-Caxfpk-PDC1t*	This work
pP6B17T6-HIS	pMTU-DO-HIS with insert *HSP150pr-Rgxfpk-PDC1t*	This work
pP6B18T6-HIS	pMTU-DO-HIS with insert *HSP150pr-Bbxfpk-PDC1t*	This work
pP6B23T6-HIS	pMTU-DO-HIS with insert *HSP150pr-Llxfpk-PDC1t*	This work
pP16A10T3-HIS	pMTU-DO-HIS with insert *SSA2pr-KmENO1-ScADH1t*	This work
pP16A26T3-HIS	pMTU-DO-HIS with insert *SSA2pr-CtPEPS-ScADH1t*	This work
pP16A27T3-HIS	pMTU-DO-HIS with insert *SSA2pr-EcppsA-ScADH1t*	This work
pP16A28T3-HIS	pMTU-DO-HIS with insert *SSA2pr-TtPEPS-ScADH1t*	This work
pP16A29T3-HIS	pMTU-DO-HIS with insert *SSA2pr-AtPPDK-ScADH1t*	This work
pP16A37T3-HIS	pMTU-DO-HIS with insert *SSA2pr-AnPEPS-ScADH1t*	This work
pP16A39T3-HIS	pMTU-DO-HIS with insert *SSA2pr-HmPEPS-ScADH1t*	This work
pB18-A10	pMTU-DO-HIS with insert *HSP150pr-Bbxfpk-PDC1t SSA2pr-KmENO1-ScADH1t*	This work
pB18-A27	pMTU-DO-HIS with insert *HSP150pr-Bbxfpk-PDC1t SSA2pr-ppsA-ScADH1t*	This work
pB18-A28	pMTU-DO-HIS with insert *HSP150pr-Bbxfpk-PDC1t SSA2pr-TtPEPS-ScADH1t*	This work
pI4-P6B18T6	pI4-MTU-DO-G418 with insert *HSP150pr-Bbxfpk-PDC1t*	This work
pI4-pP16A10T3	pI4-MTU-DO-G418 with insert *SSA2pr-KmENO1-ScADH1t*	This work
pI4-P16A26T3	pI4-MTU-DO-G418 with insert *SSA2pr-CtPEPS-ScADH1t*	This work
pI4-P16A27T3	pI4-MTU-DO-G418 with insert *SSA2pr-EcppsA-ScADH1t*	This work
pI4-P16A29T3	pI4-MTU-DO-G418 with insert *SSA2pr-AtPPDK-ScADH1t*	This work
pI4-P16A37T3	pI4-MTU-DO-G418 with insert *SSA2pr-AnPEPS-ScADH1t*	This work
pI4-P16A39T3	pI4-MTU-DO-G418 with insert *SSA2pr-HmPEPS-ScADH1t*	This work
pI4-P20A30fT7	pI4-MTU-DO-G418 with insert *FBA1pr-KmARO3*^*fbr*^-*PGK1t*	This work
pI1-B18-A27	pI1-MTU-DO-HIS with insert *HSP150pr-Bbxfpk-PDC1t SSA2pr-EcppsA-ScADH1t*	This work
pI1-B38-HIS	pI1-MTU-DO-HIS with insert *HSP150pr-Bbxfpk-PDC1t*	This work
pI4-B18-B15	pI4-MTU-DO-G418 with insert *HSP150pr-Bbxfpk-PDC1t TEF1pr-BsPta-KMXK_A03020t*	This work
pI4-B18-B20	pI4-MTU-DO-G418 with insert *HSP150pr-Bbxfpk-PDC1t TEF1pr-SePta-KMXK_A03020t*	This work
pI4-B18-B15-A27	pI4-MTU-DO-G418 with insert *HSP150pr-Bbxfpk-PDC1t TEF1pr-BsPta-KMXK_A03020t SSA2pr-EcppsA-ScADH1t*	This work
pI4-B18-B20-A27	pI4-MTU-DO-G418 with insert *HSP150pr-Bbxfpk-PDC1t TEF1pr-SePta-KMXK_A03020t SSA2pr-EcppsA-ScADH1t*	This work
pI2-B95-G418	pI2-MTU-DO-G418 with insert *HSP150pr-Bbxfpk-PDC1t TEF1pr-SePta-KMXK_A03020t FBApr-KmARO*3^*fbr*^*-PGK1t SSA2pr-ppsA-ScADH1t*	This work
pI2-B78-G418	pI2-MTU-DO-G418 with insert *HSP150pr-Bbxfpk-PDC1t TEF1pr-SePta-KMXK_A03020t FBApr-KmARO*3^*fbr*^*-PGK1t SSA2pr-KmENO1-ScADH1t*	
pI4-PEM	pI4-MTU-DO-G418 with insert *ENO1pr-ScARO10-INU1t TEF1pr-ScADH2-PGK1t*	This work
pA8-KO-P9	*TYR1pr_US-REV1pr-TYR1pr_DS* AmpR	This work
pA8-KO-P11	*TYR1pr_US-GDH2pr-TYR1pr_DS* AmpR	This work

### Production strain construction

#### Strain background engineering

For plasmid-based experiments evaluating the overexpression of *KmARO4*^*fbr*^*, KmARO7*^*fbr*^, and the shikimate and phenylalanine pathways, we used the uracil auxotrophic strain KmASR.006 as a background. To create a background strain missing wild-type *ARO3, ARO4* and *ARO7*, we sequentially inactivated the genes using our CRISPR/Cas9 plasmid pUCC001, which contains a hygromycin resistance marker. As previously described, gRNAs targeting these genes were designed using sgRNA and cloned into pUCC001 by Golden Gate assembly [50, 51]. Starting with a ura^−^ his^*−*^ auxotroph KmASR.008 we had previously constructed, we first inactivated *KmARO4* and then *KmARO7* sequentially. In each case, the CRISPR plasmid with the relevant gRNA target was transformed into KmASR.008 and selected for on YPD containing hygromycin. Up to 8 colonies were screened for mutations at *KmARO4* or *KmARO7* that caused a shift in the reading frame. After each successful gene inactivation, the strains were cured of the CRISPR plasmid by passaging an overnight culture of the confirmed mutant strain twice into YPD before being used for further genome editing. Mutant strains had their targeted genes sequenced again following curing to ensure the mutation was stable. The deletions provided us with strain KmASR.029 with an *aro4 aro7* genotype. *KmDNL4* was subsequently inactivated in this strain by CRISPR in a similar fashion to yield KmASR.039 to allow specific single integrations of pathway vectors via homologous recombination [26]. Previous characterization of NHEJ-deficient backgrounds led us to select a *dnl4-* background due to lowest variation in gene expression of reporter inserts [27]. All integrations were performed in strains derived from KmASR.039 with the *dn14-2* mutation to ensure the correct, targeted integration of single constructs. All strains used and constructed in this study are listed in Table 3, and all gRNA sequences used to inactivate genes are listed in Additional file 1: Table S4.

**Table 3:** Strains used in this study

Strain	Genotype	Source
NBRC1777	Wild-type	NITE Biological Resource Center
KmASR.006	NBRC1777 *ura3-1*	[27]
KmASR.008	NBRC1777 *ura3-1 his3-2*	[27]
KmASR.004	KmASR.006 pA1f/A4f-U	This work
KmASR.009	KmASR.006 pChor/A4f	This work
KmASR.010	KmASR.006 pAAA/Phe	This work
KmASR.021	KmASR.008 pAAA/Phe pPPP	This work
KmASR.029	KmASR.008 *aro4-1 aro7-1*	This work
KmASR.039	KmASR.029 *dnl4-2*	This work
KmASR.046	KmASR.039 *dnl4-2 aro3∆::*pI6-AAA/Phe	This work
KmASR.047	KmASR.046 *dnl4-2 lac4∆::*pI1-PPP	This work
KmASR.056	KmASR.004 pTKAL	This work
KmASR.057	KmASR.004 pPPP-ENO	This work
KmASR.058	KmASR.004 pPPP-A27	This work
KmASR.059	KmASR.004 pPPP-A28	This work
KmASR.060	KmASR.004 pPPP-A26	This work
KmASR.062	KmASR.046 *dnl4-2 lac4∆::*pI1-TKAL	This work
KmASR.063	KmASR.046 *dnl4-2 lac4∆::*pI1-TKAL-ENO	This work
KmASR.064	KmASR.046 *dnl4-2 lac4∆::*pI1-TKAL-A26	This work
KmASR.065	KmASR.046 *dnl4-2 lac4∆::*pI1-TKAL-A27	This work
KmASR.070	KmASR.004 pP6B18T6	This work
KmASR.071	KmASR.004 pB18-A27	This work
KmASR.072	KmASR.046 *dnl4-2 lac4∆::*pI1-B18-A10	This work
KmASR.074	KmASR.004 pB18-A10	This work
KmASR.075	KmASR.046 *dnl4-2 lac4∆::*pI1-B18-A27	This work
KmASR.047kd	KmASR.047 *dnl4-2 TYR1pr∆*::*GDH2pr*	This work
KmASR.047kd2	KmASR.047 *dnl4-2 TYR1pr∆*::*REV1pr*	This work
KmASR.078	KmASR.004 pB18-A28	This work
KmASR.082	KmASR.046 *dnl4-2 I4∆::*pI4-P6B18T6	This work
KmASR.085	KmASR.047 *dnl4-2 I4∆::*pI4-B18-B15-A27	This work
KmASR.086	KmASR.047 *dnl4-2 I4∆::*pI4- B18-B20-A27	This work
KmASR.087	KmASR.046 *dnl4-2 lac4∆::*pI1-P1A11T5	This work
KmASR.092	KmASR.047 *dnl4-2 I4∆::*pI4-P6B18T6	This work
KmASR.093	KmASR.047 *dnl4-2 I4∆::*pI4-B18-B15	This work
KmASR.095	KmASR.046 *dnl4-2 lac4∆::*pI1-P8A9T1	This work
KmASR.099	KmASR.004 pP6B23T6	This work
KmASR.100	KmASR.004 pP16BA27T3	This work
KmASR.101	KmASR.004 pP16BA26T3	This work
KmASR.102	KmASR.004 pP6B14T6	This work
KmASR.103	KmASR.004 pP6B17T6	This work
KmASR.107	KmASR.004 pP16BA28T3	This work
KmASR.108	KmASR.004 pP16BA29T3	This work
KmASR.109	KmASR.004 pP16BA10T3	This work
KmASR.111	KmASR.004 pP16BA37T3	This work
KmASR.112	KmASR.047 *dnl4-2 aro8∆1*	This work
KmASR.115	NBRC1777 *dnl4-1 I4∆::*pI4-PEM	This work
KmASR.117	KmASR.112 *dnl4-2 TYR1pr*::*REV1pr*	This work
KmASR.119	KmASR.062 *dnl4-2 TYR1pr*::*REV1pr aro8∆1*	This work
KmASR.120	KmASR.047 *dnl4-2 I4∆::*pI4-P16BA27T3	This work
KmASR.121	KmASR.047 *dnl4-2 I4∆::*pI4-P16BA10T3	This work
KmASR.122	KmASR.047 *dnl4-2 I4∆::*pI4-PEM	This work
KmASR.123	KmASR.062 *dnl4-2 I4∆::*pI4-PEM	This work
KmASR.127	KmASR.117 *dnl4-2 I4∆::*pI4-P16BA10T3	This work
KmASR.129	KmASR.117 *dnl4-2 I2∆::*pI2-B95-G418	This work
KmASR.141	KmASR.004 pP16BA39T3	This work
KmASR.142	KmASR.047 *dnl4-2 I4∆::*pI4-P16BA29T3	This work
KmASR.149	KmASR.046 *dnl4-2lac4∆::*pI1-B38-HIS	This work
KmASR.154	KmASR.047 *dnl4-2 I4∆::*pI4-P16BA37T3	This work
KmASR.155	KmASR.047 *dnl4-2 I4∆::*pI4-P16BA37T3	This work
KmASR.157	KmASR.047 *dnl4-2 I4∆::*pI4-P16BA39T3	This work
KmASR.158	KmASR.047 *dnl4-2 I4∆::*pI4-P20A30fT7	This work
KmASR.169	KmASR.047 *dnl4-2 I4∆::*pI4-P16BA26T3	This work
KmASR.170	KmASR.117 *dnl4-2 I2∆::*pI2-B78-G418	This work

#### Transformations and strain confirmation

All transformations into *K. marxianus* were carried out by the lithium acetate/PEG method [52]. The expression vectors were either centromeric or integrative and were constructed as recommended for the Yeast Toolkit standard using a GFP drop-out cassette whose loss confirms successful assembly and allows for green/white colony screening of *E.coli* transformed with the Golden Gate assembly. All cloning vectors used in this work have been deposited in Addgene, with their part numbers listed in Additional file 1: Table S5. For strains establishing the overexpression of the feedback-resistant shikimate or phenylalanine pathway, plasmids of interest were transformed into KmASR.008 and selected for on SD-ura agar. Subsequent plasmids containing genes to increase precursor supply (Additional file 1: Fig. S1) were transformed in the resultant strains and selected for on SD -ura- his agar. For the strain with the integrated feedback-resistant phenylalanine pathway KmASR.046, the pathway was cloned into the integrative vector pI6-MTU-DO-URA targeting *KmARO3*, resulting in the vectors pI6-AAA/Phe. To ensure efficient targeting, 850 bp sequences upstream and downstream of this gene were included as homology arms for this vector [53]. 1 µg of this plasmid was digested with FastDigest SgsI (FD1894, Fisher Scientific), transformed into KmASR.039 and selected on SD-ura agar. Colonies appearing 3–5 days after transformation were screened by duplex colony PCR: one pair of primers checking integration of the pathway, priming upstream of the *ARO3* locus and at the 5′ end of the construct, and another pair priming downstream of the *ARO3* locus and at the 3′ end of the construct. KmASR.046 was selected from colonies positive for both bands. For the overexpression of genes increasing precursor supply in KmASR.046, genes of the non-oxidative pentose phosphate pathway or phosphoketolases/phosphotransacetlyase were cloned into the vectors pI1-MTU-DO-HIS or pI4-MTU-DO-HIS with a histidine auxotrophic marker targeting the *LAC4* locus or the insertion site I4 on chromosome IV [27] respectively. Correct integration of the relevant construct was verified by colony PCR using two pairs of primers designed in a manner similar to those for checking the integration of pI6-AAA/Phe. The subsequent overexpression of genes in the resulting strains (Table 2, Additional file 1: Fig. S1) were done using the cloning vectors pI2-MTU-DO-G418 and pI4-MTU-DO-G418 that target integration sites on chromosomes V and IV respectively and previously characterised by us [27]. Primer sequences for colony PCR verification are listed in Additional file 1: Table S2. The size of the integrated constructs varied from 3820 bp (pI4-P16A10T3, to overexpress *KmENO1* in KmASR.121) to 18242 bp (pI6-AAA/Phe, to overexpress the Phe^fbr^ pathway in KmASR.046 and derivatives). The efficiency of correct integration, as evaluated from the percentage of transformants with correct colony PCR products at loci I1, I2 and I4, varied from 25–100%, and was affected by both construct size and integration locus. Efficiency was lower at the *ARO3* locus, varying from 6.25–50%.

#### Tyrosine knockdown by CRISPR/Cas9

For marker-free replacement of *TYR1pr*, a gRNA target for Cas9 covering the first 13 bases of *KmTYR1* and the first 7 upstream bases was identified by examining the start of the gene and its 5′ UTR [53]. These were used to construct gRNA plasmid pJ22. The *REV1* or *GDH2* promoters were cloned by Golden Gate assembly into a marker-free integrative vector flanked by 850 bp homology arms directly upstream and downstream of the gRNA sequence (while making sure the knock-down promoter was in-frame of *KmTYR1*) to yield plasmids pA8-KO-P9 and pA8-KO-P11 respectively. These plasmids were linearised with NotI and transformed into KmASR.047 alongside pJ22. Hygromycin-resistant colonies were screened for promoter replacement by colony PCR using a forward primer priming in the knock-down primers and a reverse primer at the end of *KmTYR1*. Promoter knockdown was 100% efficient and stable.

#### Gene deletions

gRNA targets for *KmARO8* were identified and cloned into pUCC001 as previously described. However, as these were to be transformed into an NHEJ-deficient background, it was necessary to provide a linear repair fragment for homology-directed repair. These were designed by fusing two 80 bp sequences flanking a 412 bp segment of the gene to be deleted. The repair fragment was ordered as two 90 bp oligos with a 20 bp region in common (the last 10 bp of each flanking sequence). 50 pmol of each primer was then annealed and extended by Q5 polymerase in a short PCR reaction without a template to create a 160 bp repair fragment, which was in turn transformed with the relevant gRNA plasmid targeting *KmARO8* (pJA6). Hygromycin-resistant colonies were screened by colony PCR for the deletion by primers amplifying the entire gene; the difference in PCR product size relative to an intact gene indicated a successful deletion. Ultimately *KmARO8* was knocked out in KmASR.047 to yield KmASR.112, and the deletion repeated out in the *KmTYR1* knock-down strain ASR.047kd2 to create KmASR.117.

### Production strain cultivation and sampling

The strain whose production was to be studied was grown 24 h in 5 mL of the appropriate SD medium (Formedium) at 30 °C in a shaking incubator set to 200 rpm. This culture was then diluted to an OD of 0.1 in 20 mL of minimal medium (5 g L^−1^ (NH_4_)_2_SO_4_, 3 g L^−1^ KH_2_PO_4_, 0.5 g L^−1^ MgSO_4_.7H_2_O, 20 g L^−1^ glucose with vitamins and trace elements, pH 6) [54] in a 100 mL conical flask and grown at 30 °C and 200 rpm. 1 mL samples were taken at 48 h to sample extracellular metabolites. When more time points were sampled, a smaller volume of the culture medium was taken at each time point. After the OD of the culture was measured, the culture medium was centrifuged at 13,000 rpm for 10 min and the supernatant recovered and stored at − 20 °C until analysis. For the measurement of intracellular metabolites a pellet from 2 mL culture was boiled with 75% ethanol [55], the supernatant recovered and stored at − 20 °C until analysis. All data are from biological replicates within an experiment, but the findings were validated in independent experiments (technical replicates).

### Analytical methods

Extra- and intra-cellular metabolite samples were thawed, vortexed, and transferred to screw-cap HPLC vials at an appropriate dilution. Shikimic acid, phenylalanine, tyrosine, 2-phenylethanol, tyrosol and para-hydroxyphenylacetic acid were analysed on an Agilent 1260 Infinity HPLC Infinity system with a Zorbax Eclipse Plus C18 column (4.6 × 150 mm, 3.5 µm; Agilent, Little Island, Ireland) operating at 40 °C using 20 mM KH_2_PO_4_ with 1% acetonitrile (pH 2)/acetonitrile as a solvent system at a flow rate of 0.8 mL min^−1^ as previously described [2]. The elution gradient was as follows: acetonitrile was increased from 0–10% for the first 6 min, then increased to 40% between 6 to 23 min, and then switched to 100% KH_2_PO_4_ for 23 to 27 min. All metabolites were detected using a VWD100 multi-wavelength detector measuring the absorbance at 200 nm. Data were analysed using OpenLab CDS (Agilent). The elution times of the metabolites of interest were as follows: shikimic acid, 2.32 min; tyrosine, 5.4 min; phenylalanine, 7.5 min; tyrosol/para-hydroxyphenylethanol, 10.22 min; para-hydroxyphenylacetic acid, 11.17 min; 2-phenylethanol, 17.2 min; phenylacetic acid; 17.86 min. Standards for shikimic acid, phenylalanine, tryptophan, 2-phenylethanol and phenylacetic acid were obtained from Sigma-Aldrich, whereas standards for tyrosol and para-hydroxyphenylacetic acid were obtained from Fisher Scientific. Extracellular acetate concentration was determined with an Agilent 1200 HPLC system (Agilent Technologies, CA, USA) with a REZEX 8 μL 8% H + organic acid column (300 × 7.8 mm) (Phenomenex, CA, USA) operating at 65◦C using 0.01 N H_2_SO_4_ as a solvent at a flow rate of 0.6 mL min^−1^. Organic compounds were detected with a refractive index detector. All HPLC data for aromatic compounds in the graphs can be found in Additional file 2: Supplementary Data 1.

## Supplementary information

**Additional file 1:** **Table S1.** The enzymes of the shikimate and phenylalanine/tyrosine biosynthetic pathways and the non-oxidative pentose phosphate pathway in *Kluyveromyces marxianus*. **Table S2.** Plasmids used to construct the pathway plasmids. All inserts are from *Kluyveromyces marxianus* unless specified otherwise. **Table S3.** Primers used in tTU study. Overhangs added by PCR that contain type IIS restriction enzyme sites for Golden Gate cloning are marked in boldface. **Table S4.** gRNA plasmids and their sequences used for genome engineering. The PAM is omitted. **Table S5.** Expression vectors constructed in the course of this study. Integration sites I2-I4 are described in [2]. **Figure S1.** Strain construction flowchart. Engineering strategies and strains are coloured by the use of native or heterologous enzymes, and whether an improvement in 2-phenylethanol production was observed. **Figure S2.** Extracellular acetate production for strains using **a** KmASR.004 or **b** KmASR.046 as a base. **Figure S3.** Full expression of the non-oxidative pentose pathway allows for a faster production of 2-phenylethanol over short fermentation times.** Figure S4**. The effect of knocking down* TYR1* expression and knocking out* KmARO8* on KmASR.062 results in a smaller increase in 2-PE production than when the same modifications are made in KmASR.047.** Figure S5.** Overexpressing 2-PE producing genes form the Ehrlich pathway does not significantly improve 2-PE production in* K. marxianus* NBRC1777.

**Additional file 2.** Supplementary data 1.

**Additional file 3.** Supplementary data 2.

## Data Availability

All data generated or analysed during this study are included in this published article [and its supplementary information files]. Materials used in this study are available from Addgene or from the corresponding author on reasonable request.
