# Supplementary material for: Rational engineering of Kluyveromyces marxianus to create a chassis for the production of aromatic products
Source: Microb Cell Fact. 2020 Nov 11;19:207. doi: 10.1186/s12934-020-01461-7 (PMC7659061; doi:10.1186/s12934-020-01461-7)
Supplement: Supplementary file 1 — Additional file 1: Table S1. The enzymes of the shikimate and phenylalanine/tyrosine biosynthetic pathways and the non-oxidative pentose phosphate pathway in Kluyveromyces marxianus. Table S2. Plasmids used to construct the pathway plasmids. All inserts are from Kluyveromyces marxianus unless specified otherwise. Table S3. Primers used in tTU study. Overhangs added by PCR that contain type IIS restriction enzyme sites for Golden Gate cloning are marked in boldface. Table S4. gRNA plasmids and their sequences used for genome engineering. The PAM is omitted. Table S5. Expression vectors constructed in the course of this study. Integration sites I2-I4 are described in [2]. Figure S1. Strain construction flowchart. Engineering strategies and strains are coloured by the use of native or heterologous enzymes, and whether an improvement in 2-phenylethanol production was observed. Figure S2. Extracellular acetate production for strains using a KmASR.004 or b KmASR.046 as a base. Figure S3. Full expression of the non-oxidative pentose pathway allows for a faster production of 2-phenylethanol over short fermentation times. Figure S4. The effect of knocking down TYR1 expression and knocking out KmARO8 on KmASR.062 results in a smaller increase in 2-PE production than when the same modifications are made in KmASR.047. Figure S5. Overexpressing 2-PE producing genes form the Ehrlich pathway does not significantly improve 2-PE production in K. marxianus NBRC1777. [file 12934_2020_1461_MOESM1_ESM.docx]

Additional File 1 for

**Rational engineering of *Kluyveromyces marxianus* to create a chassis for the production of aromatic products**

Arun S. Rajkumar and John P. Morrissey

School of Microbiology / Centre for Synthetic Biology and Biotechnology / Environmental Research Institute / APC Microbiome Institute, University College Cork, Ireland

**Table S1.** The enzymes of the shikimate and phenylalanine/tyrosine biosynthetic pathways and the non-oxidative pentose phosphate pathway in *Kluyveromyces marxianus*. The sequence identity was determined by BLASTing the translated genetic sequence against the reference genome of *Saccharomyces cerevisiae* S288C. Sequence identity with *S. cerevisiae* orthologues, where they exist, is also provided.

| **Protein** | **Gene locus in the NBRC1777 genome** | ***S. cerevisiae* paralogue/Sequence identity** | **ORF size (bp)** |
| --- | --- | --- | --- |
| KmAro3 | KMAR_40115 | Aro3/79.7% | 1119 |
| KmAro4 | KMAR_20396 | Aro4/85.5% | 1095 |
| KmAro1 | KMAR_40172 | Aro1/71.3% | 4743 |
| KmAro2 | KMAR_80097 | Aro2/86.4% | 1131 |
| KmAro7 | KMAR_80320 | Aro7/75.8% | 780 |
| KmPha2 | KMAR_70145 | Pha2/48.8% | 936 |
| KmTyr1 | KMAR_10786 | Tyr1/72.7% | 1329 |
| KmAro8 | KMAR_20249 | Aro8/62.2%; 29.9% with Aro9 | 1494 |
| KmAro9 | KMAR_60415 | Aro9/42.7%; 28.5% with Aro8 | 1593 |
| KmAro10 | KMAR_20565 | Aro10/48.5% | 1893 |
| KmTkl1 | KMAR_80293 | Tkl1/77.9%; 71.7% with Tkl2 | 2040 |
| KmTal1 | KMAR_30605 | Tal1/77.9%; 70% with Nqm2 | 1005 |
| KmRpe1 | KMAR_60028 | Rpe1/71.7% | 711 |
| KmRki1 | KMAR_80221 | Rki1/73% | 819 |
| KmEno1 | KMAR_10447 | Eno1/86%;86% with Eno2 | 1314 |
| Aromatic aminotransferase II | KMAR_50141 | 45% identity with Aro8; 19% identity with Aro9 | 1434 |

**Table S2.** Plasmids used to construct the pathway plasmids. All inserts are from *Kluyveromyces marxianus* unless specified otherwise.

| **Plasmid** | **Features** | **Comments/Reference** | **Insert size (bp)** |
| --- | --- | --- | --- |
| Part storage vectors | | | |
| pYTK001 | Cloning vector for storage, CamR | [1]; Addgene ref. 65108; part of kit 1000000061 | n/a |
| pKmK.P1 | pYTK001 *PGK1pr* | [2];Addgene ref. 125034 | 740 |
| pKmK.P2 | pYTK001 *PDC1pr* | [2]; Addgene ref. 125035 | 999 |
| pKmK.P3 | pYTK001 *ENO1pr* | [2]; Addgene ref. 125036 | 725 |
| pKmK.P5 | pYTK001 *HSP150pr* | [2]; Addgene ref. 125038 | 999 |
| pKmK.P7 | pYTK001 *TEF1pr* | [2]; Addgene ref. 125040 | 874 |
| pKmK.P8 | pYTK001 *REV1pr* | [2]; Addgene ref. 125041 | 979 |
| pKmK.P10 | pYTK001 *GDH2pr* | [2]; Addgene ref. 125043 | 994 |
| pKmK.P12 | pYTK001 *TSA1pr* | [2]; Addgene ref. 125045 | 967 |
| pKmK.P15 | pYTK001 *TDH3pr* | [2]; Addgene ref. 125048 | 534 |
| pKmK.T1 | pYTK001 *INU1t* | [2]; Addgene ref. 125053 | 250 |
| pKmK.T2 | pYTK001 *LAC4t* | [2]; Addgene ref. 125054 | 250 |
| pKmK.T3 | pYTK001 *KMXK_A03020t* | [2]; Addgene ref. 125055 | 252 |
| pKmK.T4 | pYTK001 *PDC1t* | [2]; Addgene ref. 125056 | 250 |
| pYTK053 | pYTK001 *ScADH1t* | [1]; Addgene ref. 65160; part of kit 1000000061 | 225 |
| pYTK054 | pYTK001 *ScPGK1t* | [1]; Addgene ref. 65161; part of kit 1000000061 | 225 |
| pA1f | pYTK001 *ARO4^K221L^* | This work | 1095 |
| pA2 | pYTK001 *ARO1* | This work | 4743 |
| pA3 | pYTK001 *ARO2* | This work | 1131 |
| pA4f | pYTK001 *ARO4^G141S^* | This work | 780 |
| pA5 | pYTK001 *PHA2* | This work | 936 |
| pA7 | pYTK001 *ARO9* | This work | 1893 |
| pA8 | pYTK001 *TYR1* | This work | 1329 |
| pA9 | pYTK001 *TKL1* | This work | 2040 |
| pA11 | pYTK001 *TAL1* | This work | 1005 |
| pA19 | pYTK001 *RPE1* | This work | 711 |
| pA20 | pYTK001 *RKI1* | This work | 819 |
| pA10 | pYTK001 *ENO1* | This work | 1314 |
| pA26 | pYTK001 *CtPEPS* | This work | 2406 |
| pA27 | pYTK001 *ppsA* | This work | 2379 |
| pA28 | pYTK001 *TtPEPS* | This work | 2409 |
| pA29 | pYTK001 *AtPPDK* | This work | 2628 |
| pA30f | pYTK001 *KmARO3^K222L^* | This work | 1119 |
| pA37 | pYTK001 *AnPEPS* | This work | 2391 |
| pA39 | pYTK001 *HmPEPS* | This work | 2268 |
| pB14 | pYTK001*Caxfpk* | This work | 2391 |
| pB15 | pYTK001*Bspta* | This work | 972 |
| pB17 | pYTK001*Rgxfpk* | This work | 2511 |
| pB18 | pYTK001*Bbxfpk* | This work | 2478 |
| pB20 | pYTK001*Septa* | This work | 1019 |
| pB23 | pYTK001*Llxfpk* | This work | 2469 |
| pI6L | pYTK001 with left homology arm for targeting *ARO3* | This work | 850 |
| pI6R | pYTK001 with right homology arm for targeting *ARO3* | This work | 850 |
| Expression cassette/transcriptional unit (TU)-containing plasmids. Cassettes are listed as promoter (*pr*) – gene – terminator (*t*). They are not in expression vectors for *K. marxianus* | | | |
| pP2A1fT3-TU | *PDC1pr-ARO4fbr-ScADH1t* AmpR | This work | 2689 |
| pP13A2T5-TU | *TSA1pr-ARO1-KMXK_A03020t* AmpR | This work | 6346 |
| pP1A3T4-TU | *PGK1pr-ARO2-LAC4t* AmpR | This work | 2494 |
| pP8A4fT6-TU | *TEF1pr-ARO7fbr-PDC1t* AmpR | This work | 2259 |
| pP3A5T1-TU | *ENO1pr-PHA2-INU1t* AmpR | This work | 2281 |
| pP6A7T2-TU | *HSP150pr-ARO9-ScPGK1t* AmpR | This work | 3088 |
| pP8A9T1-TU | *TEF1pr-TKL1-INU1t* AmpR | This work | 3485 |
| pP1A11T5-TU | *PGK1pr-TAL1-MXK_A03020t* AmpR | This work | 2365 |
| pP19A19T6-TU | *TDH3pr-RPE1-PDC1t* AmpR | This work | 1865 |
| pP13A20T2-TU | *TSA1pr-RKI1-ScPGK1t* AmpR | This work | 2347 |
| pP6B18T6-TU | *HSP150pr-Bbxfpk-PDC1t* AmpR | This work | 5558 |
| pP16A10T3-TU | *SSA2pr-KmENO1-ScADH1t* AmpR | This work | 2701 |
| pP16A26T3-TU | *SSA2pr-CtPEPS-ScADH1t* AmpR | This work | 3820 |
| pP16A27T3-TU | *SSA2pr-ppsA-ScADH1t* AmpR | This work | 3793 |
| pP16A28T3-TU | *SSA2pr-TtPEPS-ScADH1t* AmpR | This work | 3796 |
| pP8B15T5-TU | *TEF1pr-Bspta-KMXK_A03020t* AmpR | This work | 2485 |
| pP8B20T5-TU | *TEF1pr-Septa-KMXK_A03020t* AmpR | This work | 2530 |
| pP20A30fT7-TU | *FBA1pr-KmARO3^fbr^*-*PGK1t* AmpR | This work | 2662 |
| pP3A25T1-TU | *ENO1pr-ScARO10-INU1t* AmpR | This work | 4884 |
| pP8A38T7-TU | *TEF1pr-ScADH2-PGK1t* AmpR | This work | 2494 |
|  |  |  |  |

**Table S3.** Primers used in tTU study. Overhangs added by PCR that contain type IIS restriction enzyme sites for Golden Gate cloning are marked in boldface.

| **Primer** | **Sequence (5’ to 3’)** | **Description** |
| --- | --- | --- |
| ASR_A1F | **GCATCGTCTCATCGGTCTCATATG**TCAGCTACACCACAACCTAT | Forward primer for *KmARO4* |
| ASR_A1MR | **CACGTCTCAGAAG**GGTAACACCCATGAAGTGATG | Reverse primer for adding K221L mutation; used with ASR_A1F |
| ASR_A1MF | **TTCGTCTCACTTC**ACGGTGTTGCTGCCATCA | Forward primer for adding K221L mutation; used with ASR_A1R |
| ASR_A1R | **ATGCCGTCTCAGGTCTCAGGATCTA**TTTAGCGGCCTTCTTTTTTAGTTCT | Reverse primer for *KmARO4* |
| ASR_A2F | **GCATCGTCTCATCGGTCTCATATG**TCCGTTGAATTGTCCAAA | Forward primer for *KmARO1* |
| ASR_A2MR | **CACGTCTCTAGTT**TCAAATTTATCAAAGTTATGGGGC | Reverse primer for eliminating an internal BsaI site; used with ASR_A2F |
| ASR_A2MF | **TTCGTCTCTAACT**GACGATATCGAGCAAGTTAAGAAA | Reverse primer for eliminating an internal BsaI site; used with ASR_A2F |
| ASR_A2R | **ATGCCGTCTCAGGTCTCAGGATCTA**AACTTCATTCGTAACTGCTTCA | Reverse primer for *KmARO1* |
| ASR_A3F | **GCATCGTCTCATCGGTCTCATATG**TCCACCTTTGGTCAAATTTTC | Forward primer for *KmARO2* |
| ASR_A3R | **ATGCCGTCTCAGGTCTCAGGATCTA**TGAAACGATAGAGAAAGCGG | Reverse primer for *KmARO2* |
| ASR_A4F | **GCATCGTCTCATCGGTCTCATATG**GATTTTTTTAAACCAGAAACTGTTCT | Forward primer for *KmARO7* |
| ASR_A4MR | **CACGTCTCACCTT**AATTGAGATTGCACAATTTCCA | Reverse primer for eliminating an internal BsmBI site; used with ASR_A4F |
| ASR_A4MF | **TTCGTCTCAAAGG**CGGTTCGAGTCACCAGAC | Forward primer for eliminating an internal BsmBI site; used with ASR_A4R |
| ASR_A4R | **ATGCCGTCTCAGGTCTCAGGATCTA**TTTCTCTTCATCCTCCAACCTC | Reverse primer for *KmARO7* |
| ASR_A4M2R | **CACGTCTCATAGA**AAAATTCTCAGATGTGTTTCCC | Reverse primer for adding a G141S mutation; used with ASR_A4F |
| ASR_A4M2F | **TTCGTCTCATCTA**TATCCCTCGTAGCCACAC | Forward primer for adding a G141S mutation; used with ASR_A4R |
| ASR_A5F | **GCATCGTCTCATCGGTCTCATATG**GTTAAAGTGCTGTATCTAGGG | Forward primer for *KmPHA2* |
| ASR_A5MR | **CACGTCTCATCGT**GAGGAAATAGAACACATATTTAACC | Reverse primer for eliminating an internal BsaI site; used with ASR_A5F |
| ASR_A5MF | **TTCGTCTCAACGA**CCGTTCCATGCGGACTCC | Forward primer for eliminating an internal BsaI site; used with ASR_A5R |
| ASR_A5R | **ATGCCGTCTCAGGTCTCAGGATCTA**AGACACCTGGTAATACGAAGGA | Reverse primer for *KmPHA2* |
| ASR_A7F | **GCATCGTCTCATCGGTCTCATATG**GTCGTGAAGATTGATGATAAGAC | Forward primer for *KmARO9* |
| ASR_A7R | **ATGCCGTCTCAGGTCTCACCTA**GTTTTTATACTCTTTGAAAAATCTTTCA | Reverse primer for *KmARO9* |
| ASR_A8F | **GCATCGTCTCATCGGTCTCATATG**ATTGCAACTGAGGAACAGAT | Forward primer for *KmTYR1* |
| ASR_A8R | **ATGCCGTCTCAGGTCTCAGGATCTA**ATCCTTGGAATGTTGAAGTATCG | Reverse primer for *KmTYR1* |
| ASR_A9F | **GCATCGTCTCATCGGTCTCATATG**TCTCAATATTCCGATATCGATCGT | Forward primer for *KmTKL1* |
| ASR_A9MR | **CACGTCTCATACA**AAGTTCAAGAAAGTACCACCGTA | Reverse primer for eliminating internal BsaI and BsmBI sites; used with ASR_A9F |
| ASR_A9MF | **TGCGTCTCTTGTA**TCTTACGCTGCAGGTGCA | Forward primer for a synthetic version of the last 740 bases of *KmTKL1* with BsaI and BsmBI sites eliminated; used with ASR_A9R |
| ASR_A9R | **ATGCCGTCTCAGGTCTCAGGATCTA**GAAAGCAGTGTTCAAAGGAGAATA | Reverse primer for *KmTKL1* |
| ASR_A11F | **GCATCGTCTCATCGGTCTCATATG**TCTGAACCAGCTGCTAAG | Forward primer for *KmTAL1* |
| ASR_A11R | **ATGCCGTCTCAGGTCTCAGGATCTA**AGCTTGGATCTTAGCCTT | Reverse primer for *KmTAL1* |
| ASR_A19F | **GCATCGTCTCATCGGTCTCATATG**GTCCAACCTATCATTGCTCCTT | Forward primer for *KmRPE1* |
| ASR_A19R | **ATGCCGTCTCAGGTCTCACCTATG**CCAAGAGGTCTTTGGC | Reverse primer for *KmRPE1* |
| ASR_A20F | **GCATCGTCTCATCGGTCTCATATG**TACTGTGCTGTAAGCAGGCGTGTTC | Forward primer for *KmRKI1* |
| ASR_A20MR | **CACGTCTCA**ATCCGTAACCACGGGCCCCGCT | Reverse primer for eliminating an internal BsmBI site; used with ASR_A20F |
| ASR_A20MF | **TTCGTCTCA**GGATAACTGCAACTTCATCATTGAC | Forward primer for eliminating an internal BsmBI site; used with ASR_A20R |
| ASR_A20R | **ATGCCGTCTCAGGTCTCACCTA**CAACACCTGCAGCTCGAC | Reverse primer for *KmRKI1* |
| ASR_I6LF_MTU | **GCATCGTCTCATCGGTCTCACCCTTTCAGGCGCGCC**TAAGGCAGTAGAGCAGTAG | Forward primer for left homology arm targeting *KmARO3*; used in pI6-MTU-DO-URA |
| ASR_I  6LR_MTU | **ATGCAGGTCTCACGTTCGTCTCATCAGTCTAGATGCGAATTC**TACACTTATTACCGTTAGTTACCTAT | Reverse primer for left homology arm targeting *KmARO3*; used in pI6-MTU-DO-URA. |
| ASR_I6RFbis | **GCATCGTCTCATCGGTCTCAGAGT**AGGCTAAGGTTGTTGATGC | Forward primer for right homology arm targeting *KmARO3*; used in pI6-MTU-DO-URA.^a^ |
| ASR_I6RRbis | **ATGCCGTCTCAGGTCTCATCGGCTGAGGCGCGCCAA**CAACACTATTACAACTGATTCTACA | Reverse primer for right homology arm targeting *KmARO3*; used in pI6-MTU-DO-URA |
| ASR_A8_US_F | **GCATCGTCTCATCGGTCTCACCCT**TGGGTGCTTTTCAAGCAC | Forward primer for left homology arm targeting *TYR1pr*. |
| ASR_A8_US_R | **GCATCGTCTCATCGGTCTCACGTT**TGTGCTGAATAAAGTAGTATTGTTATAC | Reverse primer for left homology arm targeting *TYR1pr*. |
| ASR_A8KO_DS_R | **ATGCCGTCTCAGGTCTCATCGG**AGCTACGGAAAGTCTTTCAG | Reverse primer for right homology arm targeting *TYR1pr*, used with ASR_A8F |
| ASR_K1F | TTTGCTGGCCTTTTGCTC | General colony PCR forward primer, priming at the 3' end of ColE1 in all of plasmid backbones; used for confirmation of plasmid assembly and sequencing of level I plasmid inserys |
| ASR_K2R | CATCTGGATTTGTTCAGAACG | Colony PCR reverse primer, priming downstream of the BsmBI cloning site in YTK001; used for confirmation of plasmid assembly and sequencing of level I plasmid inserts |
| ASR_K1R | ATTGGTAACTGTCAGACCAAGTTTA | Colony PCR reverse primer, priming at the 5' end of AmpR; used for confirmation of level II plasmid assembly |
| ASR_K15R | GTATACATGCATTTACTTATAATACAGT | Reverse primer for checking plasmid assembly; primes in the 3’ end of the *URA3* marker |
| ASR_K17R | GTATGCAGCAGCTTTAAATAAT | Reverse primer for checking plasmid assembly; primes in the 3’ end of the *HIS3* marker |
| ASR_K15F | ACTGTATTATAAGTAAATGCATGTATAC | Forward primer for checking integration of vector; primes in the 3’ end of the *URA3* marker |
| ASR_K17F | ATTATTTAAAGCTGCTGCATAC | Forward primer for checking integration of vector; primes in the 3’ end of the *HIS3* marker |
| ASR_K11F | AGCTCTGCCATTATTTGATCTAGG | Forward primer for checking integration of vector; primes in the 3’ end of the *KanMX* marker |
| ASR_I1US_F | CATTAGAACCTTTTTCAACACTC | Forward primer for checking integration at I1/*LAC4* integration site; primes 92bp upstream of the site. |
| ASR_I1DS_R | CTTAGTGGTTGTGAAGGTTT | Reverse primer for checking integration at I1/*LAC4* integration site; primes 90bp downstream of the site. |
| ASR_I3_US_F | GTTTCGAAAAGGGTTTCGA | Forward primer for checking integration at I2 integration site; primes 80bp upstream of the site. |
| ASR_I3_DS_R | CTGCTGAAACAACATCACC | Reverse primer for checking integration at I2 integration site; primes 81bp downstream of the site. |
| ASR_I5_US_F | AGTAGTGAGTGACAGACAC | Forward primer for checking integration at I4 integration site; primes 62bp upstream of the site. |
| ASR_I5_DS_R | GCAGTTTCTGTGCAGAAAA | Reverse primer for checking integration at I4 integration site; primes 102bp downstream of the site. |
| ASR_I6_US_F | AGCCTGTCTACGTACAAC | Forward primer for checking integration at I6/*ARO3* integration site; primes 107bp upstream of the site. |
| ASR_I6_DS_R | CGTTCCAAGGAAACCACTATA | Reverse primer for checking integration at I6/*ARO3* integration site; primes 98bp upstream of the site. |

^a^The primer contains a 5’ overhang that contains a BsmBI site to make it compatible for a multigene integrative vector according to the Yeast Toolkit standard.

**Table S4.** gRNA plasmids and their sequences used for genome engineering. The PAM is omitted.

| Target | Sequence (5’ to 3’) | Remarks |
| --- | --- | --- |
| JA1/*KmARO4* | TTCTCTGTGCAATTGAGACT | Targets a predicted structural element in Aro4p; created a frameshift mutation across codons 177 and 178 |
| JA4/*KmARO7* | AATAGGACGCCAGAATCTTG | Targets a conserved region in Aro7p; deleted codon 105 and created a frameshift in codon 106 |
| JA6/*KmARO8* | TGAAGGGCGTGTGATAAGAA | Targets a region near the predicted active site |
| JA22/*KmTYR1pr* | CAGCACAATGATTGCAACTG |  |

**Table S5.** Expression vectors constructed in the course of this study. Integration sites I2-I4 are described in [2].

| Name | Description | Addgene ID |
| --- | --- | --- |
| pMTU-DO-URA | Low-copy expression vector, uracil selection | 160004 |
| pMTU-DO-HIS | Low-copy expression vector, histidine selection | 160005 |
| pMTU-DO-LEU | Low-copy expression vector, leucine selection | 160006 |
| pMTU-DO-G418 | Low-copy expression vector, G418 selection | 160007 |
| pMTU-DO-HPH | Low-copy expression vector, hygromycin selection | 160008 |
| pI1-MTU-DO-URA | Integrative vector targeting the *LAC4* locus, uracil selection. | 160009 |
| pI1-MTU-DO-HIS | Integrative vector targeting the *LAC4* locus, histidine selection. | 160142 |
| pI1-MTU-DO-LEU | Integrative vector targeting the *LAC4* locus, leucine selection. | 160013 |
| pI1-MTU-DO-G418 | Integrative vector targeting the *LAC4* locus, G418 selection. | 160012 |
| pI1-MTU-DO-HPH | Integrative vector targeting the *LAC4* locus, hygromycin selection. | 160014 |
| pI2-MTU-DO-URA | Integrative vector targeting integration site I2, uracil selection. | 160015 |
| pI2-MTU-DO-HIS | Integrative vector targeting integration site I2, histidine selection. | 160016 |
| pI2-MTU-DO-LEU | Integrative vector targeting integration site I2, leucine selection. | 160017 |
| pI2-MTU-DO-G418 | Integrative vector targeting integration site I2, G418 selection. | 160018 |
| pI2-MTU-DO-HPH | Integrative vector targeting integration site I2, hygromycin selection | 160019 |
| pI3-MTU-DO-URA | Integrative vector targeting integration site I3, uracil selection. | 160020 |
| pI3-MTU-DO-HIS | Integrative vector targeting integration site I3, histidine selection. | 160021 |
| pI3-MTU-DO-LEU | Integrative vector targeting integration site I3, leucine selection. | 160176 |
| pI3-MTU-DO-G418 | Integrative vector targeting integration site I3, G418 selection. | 160023 |
| pI3-MTU-DO-HPH | Integrative vector targeting integration site I3, hygromycin selection | 160207 |
| pI4-MTU-DO-URA | Integrative vector targeting integration site I4, uracil selection. | 160025 |
| pI4-MTU-DO-HIS | Integrative vector targeting integration site I4, histidine selection. | 160026 |
| pI4-MTU-DO-LEU | Integrative vector targeting integration site I4, leucine selection. | 160029 |
| pI6-MTU-DO-URA | Integrative vector targeting the *ARO3* locus, uracil selection. | 160030 |
| pI4-MTU-DO-G418 | Integrative vector targeting integration site I4, G418 selection. | 160215 |
| pI4-MTU-DO-HPH | Integrative vector targeting integration site I4, hygromycin selection | 160216 |
| pMTU-DO-URA-C4 | High-copy expression vector, uracil selection | 160339 |
| pMTU-DO-HIS-C4 | High-copy expression vector, histidine selection | 160340 |
| pMTU-DO-LEU-C4 | High-copy expression vector, leucine selection | 160341 |
| pMTU-DO-G418-C4 | High-copy expression vector, G418 selection | 160342 |
| pMTU-DO-HPH-C4 | High-copy expression vector, hygromycin selection | 160343 |

| 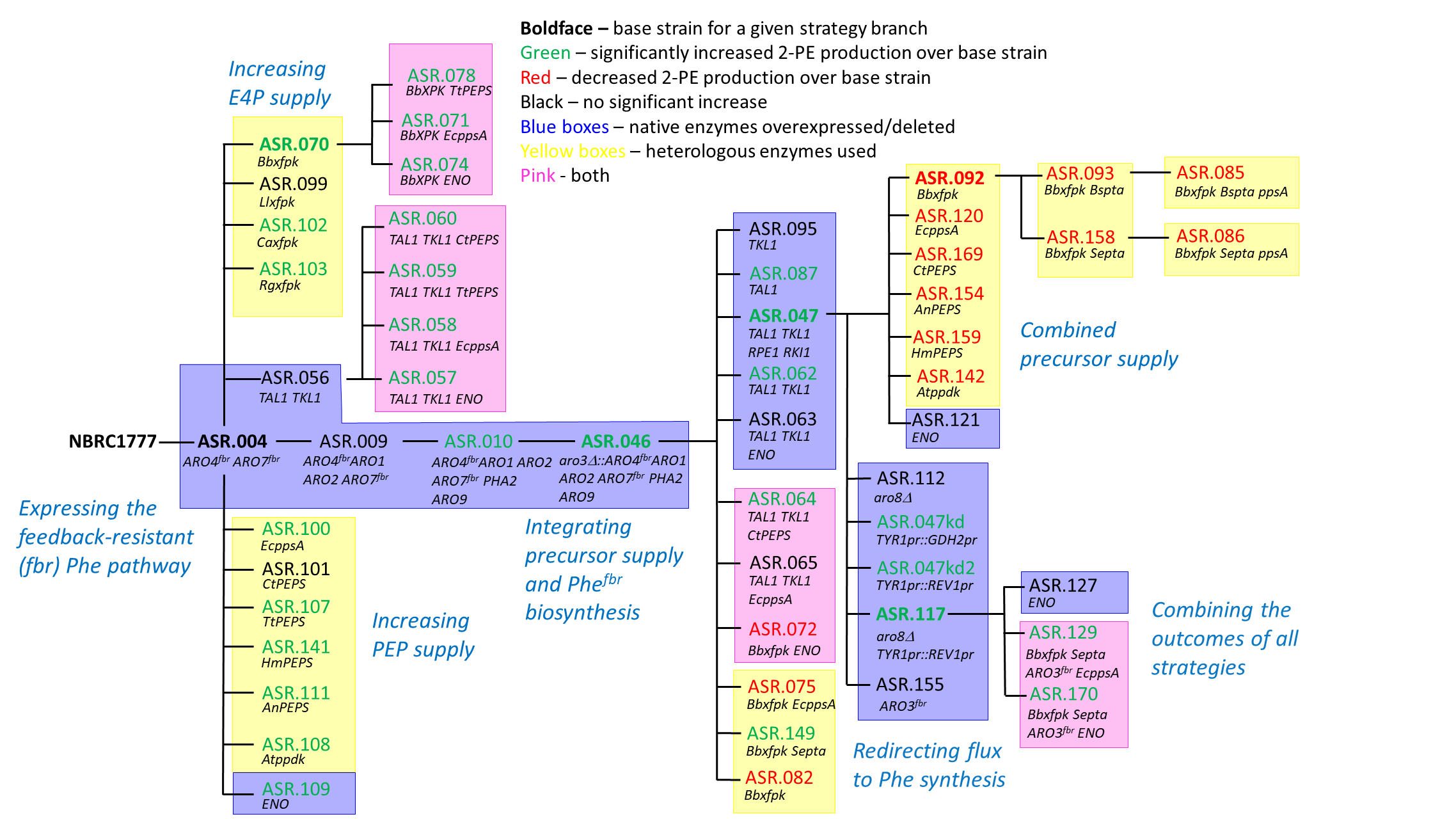 |
| --- |
| **Figure S1.** Strain construction flowchart. Engineering strategies and strains are coloured by the use of native or heterologous enzymes, and whether an improvement in 2-phenylethanol production was observed. |

| 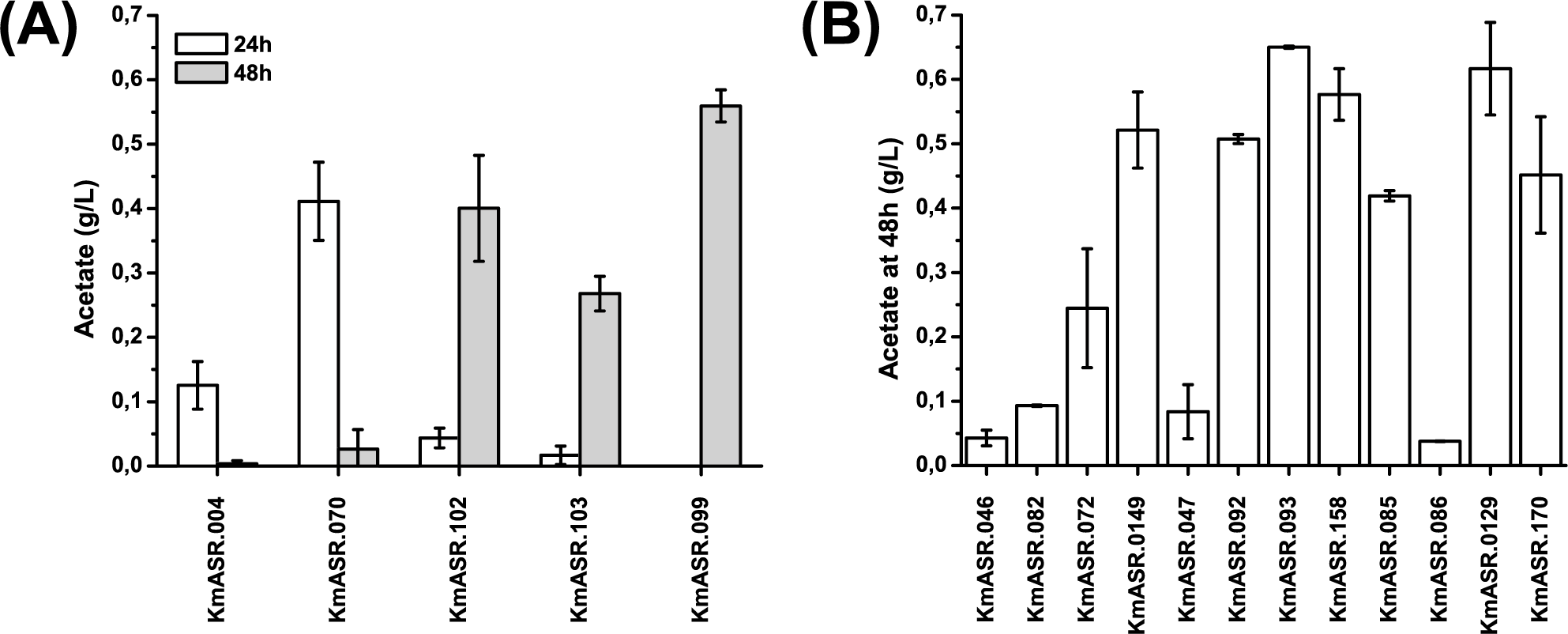 |
| --- |
| **Figure S2.** Extracellular acetate production for strains using (A) KmASR.004 or (B) KmASR.046 as a base. Strain descriptions are provided in Figure S1 and Table 3. Data are plotted as the mean ± s.d. of at least three biological replicates. |

| 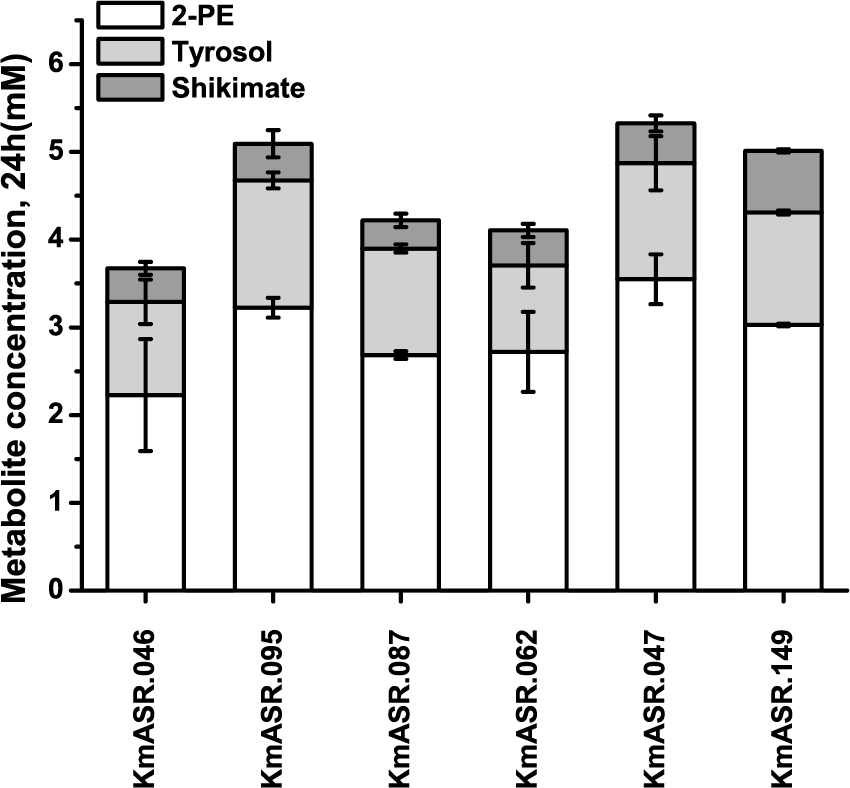 |
| --- |
| **Figure S3.** Full expression of the non-oxidative pentose pathway allows for a faster production of 2-phenylethanol over short fermentation times. While overexpressing *TKL1* (ASR.095) produces nearly as much 2-PE as when the entire non-oxidative PPP is overexpressed (ASR.047), little more is produced over the next 24h (Figure 4B), which aided us in our decision to select ASR.047 as a chassis for further engineering efforts. Data are plotted as mean ± s.d. of at least 3 biological replicates. Figure 4B in the main text provides more information on the genes overexpressed in each strain. |

| 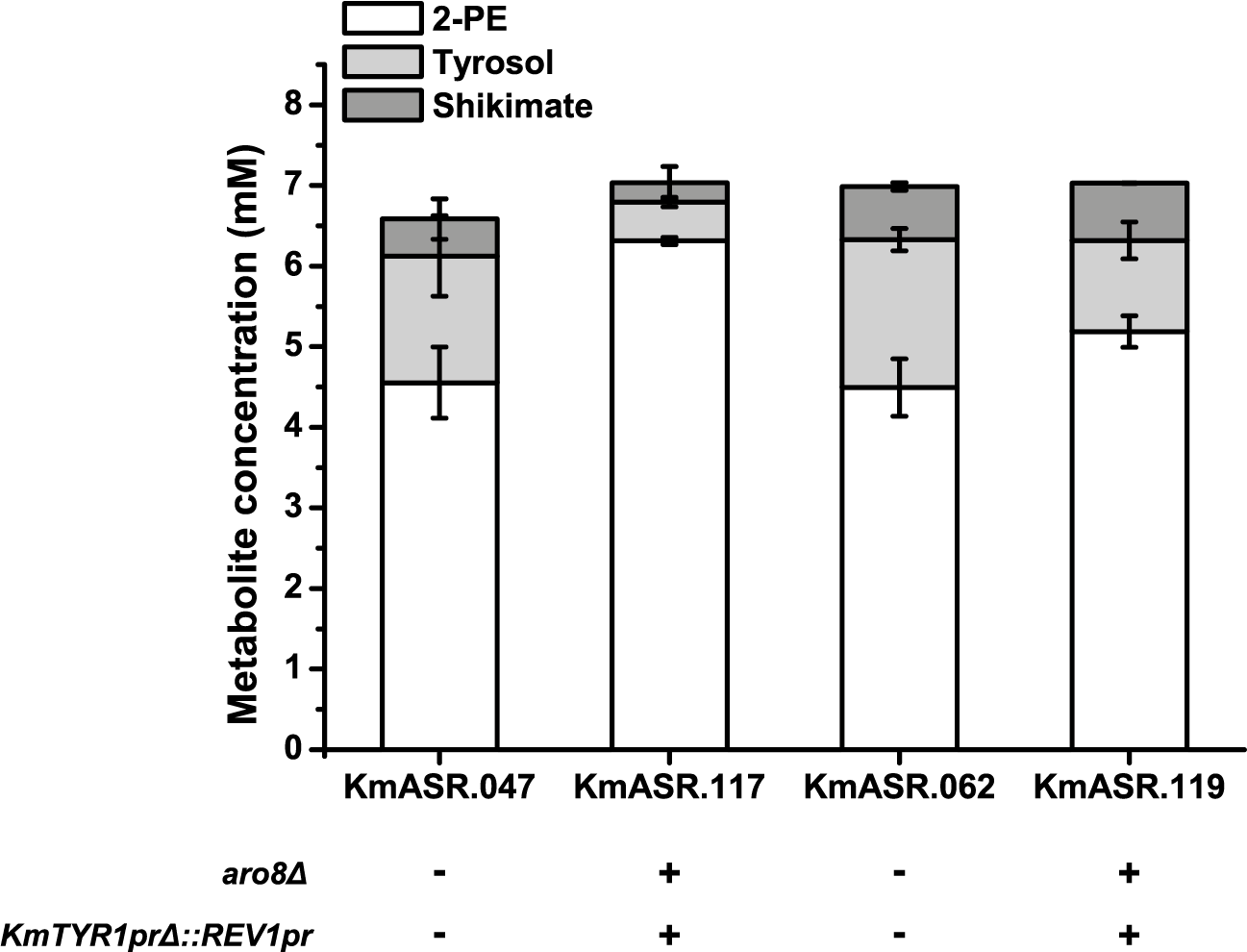 |
| --- |
| **Figure S4.** The effect of knocking down *TYR1* expression and knocking out *KmARO8* on KmASR.062 results in a smaller increase in 2-PE production than when the same modifications are made in KmASR.047. Full strain descriptions are provided in Figure S1 and Table 3, and data are plotted as the mean ± s.d. of at least three replicates. |

| 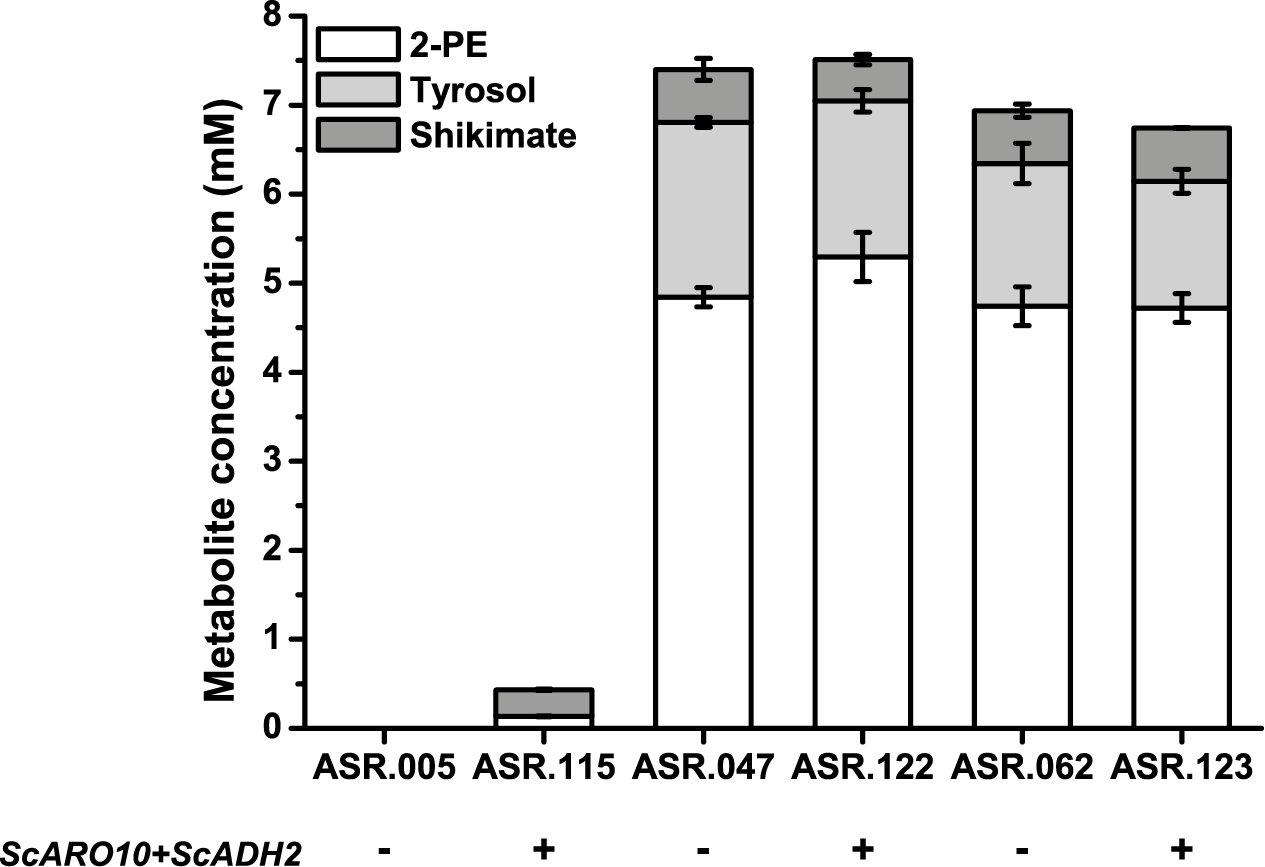 |
| --- |
| **Figure S5.** Overexpressing 2-PE producing genes form the Ehrlich pathway does not significantly improve 2-PE production in *K. marxianus* NBRC1777. The same modifications were used in a wild-type *K. marxianus* DMKU3-1042 to overproduce 1.3 g/L, or over 10mM 2-PE after 72h culture [3]. The same genes were cloned and overexpressed in two phenylalanine/2-PE overproducing strains, KmASR.047 and KmASR.062, as well as wild-type NBRC1777, and cultured for 72h in shake flasks as in ref. 3. Full strain descriptions are provided in Figure S1 and Table 33. Data are plotted as the mean ± s.d. of duplicates. |

**SUPPLEMENTARY REFERENCES**

1. Lee ME, DeLoache WC, Cervantes B, Dueber JE. A Highly Characterized Yeast Toolkit for Modular, Multipart Assembly. ACS Synth Biol. 2015;4:975–86.

2. Rajkumar AS, Varela JA, Juergens H, Daran JMG, Morrissey JP. Biological parts for *Kluyveromyces marxianus* synthetic biology. Front Bioeng Biotechnol. 2019;7:1–15.

3. Kim TY, Lee SW, Oh MK. Biosynthesis of 2-phenylethanol from glucose with genetically engineered *Kluyveromyces marxianus*. Enzyme Microb Technol. Elsevier Inc.; 2014;61–62:44–7.
